# MYSM1-mediated epigenetic modification dysregulation leads to immunosuppression and secondary infections in sepsis

**DOI:** 10.1371/journal.ppat.1013935

**Published:** 2026-02-12

**Authors:** Jiali Xiong, Xin Cheng, Xiaoxing Xiong, Heyang Zhang, Qi An, Zhiqiang Li, Hong Fan, Guangli Li, Wei Li, Mingfu Tian, Jingjun Lv

**Affiliations:** 1 Department of Emergency, Renmin Hospital of Wuhan University, Wuhan, Hubei, PR China; 2 Department of Neurosurgery, Renmin Hospital of Wuhan University, Wuhan, Hubei, PR China; 3 Department of Gastroenterology, Beijing Friendship Hospital, Capital Medical University, Beijing, PR China; 4 Department of Oncology, Renmin Hospital of Wuhan University, Wuhan, Hubei, PR China; 5 Department of Clinical Laboratory, Institute of Translational Medicine, Renmin Hospital of Wuhan University, Wuhan, Hubei, PR China; 6 Postgraduate Training Base at Shanghai Gongli Hospital, Ningxia Medical University, Shanghai, PR China; 7 Department of Anesthesiology, Renmin Hospital of Wuhan University, Wuhan, Hubei, PR China; 8 State Key Laboratory of Virology, College of Life Sciences, Wuhan University, Wuhan, Hubei, PR China; INSERM, FRANCE

## Abstract

Sepsis is a life-threatening condition characterized by a dysregulated immune response to infection, often leading to organ dysfunction and even death. During the recovery phase of sepsis, patients frequently exhibit impaired antimicrobial function of immune cells, which exacerbates the state of immunosuppression and increases the risk of secondary infections. However, therapeutic strategies targeting sepsis-induced immunosuppression have yet to achieve breakthrough progress, with the core challenge lying in the significant gaps in understanding the molecular mechanisms underlying immunosuppression. In this study, we integrated clinical samples, mouse models, and molecular mechanisms to reveal that the reduction in macrophage function and epigenetic dysregulation, particularly histone ubiquitination, are central drivers of sepsis-induced immunosuppression. Further investigation demonstrated that MYSM1, a deubiquitinase, plays a pivotal role in regulating this ubiquitination process. Targeted deletion of the N-terminal domain of MYSM1 markedly enhances the inflammatory response during the early phase of secondary infection in sepsis, facilitating bacterial clearance and significantly mitigating tissue damage in the late phase of secondary infection, thereby improving the survival outcomes in mice. Overall, our study elucidates the role of MYSM1-mediated dysregulation of epigenetic modifications in the immune response during the late phase of sepsis, providing a novel therapeutic approach for addressing sepsis-related immune dysfunction.

## Author summary

Sepsis is a severe and often fatal condition caused by an uncontrolled immune response to infection. Although early treatment can control the initial inflammation, many patients enter a stage of immune paralysis during recovery, which makes them highly susceptible to secondary infections. The mechanisms driving this immunosuppressive state remain poorly understood, hindering the development of effective therapies. In this study, we combined clinical samples, mouse models, and molecular experiments to uncover how the histone deubiquitinase MYSM1 contributes to sepsis-induced immunosuppression. We found that MYSM1-mediated dysregulation of histone ubiquitination leads to impaired macrophage antimicrobial function and weakened immune responses. Deletion of the N-terminal domain of MYSM1 restored macrophage activity, enhanced bacterial clearance, and improved survival in septic mice, demonstrating that MYSM1 is a key regulator of immune reprogramming during sepsis. These findings provide new insights into the epigenetic mechanisms underlying sepsis-induced immune dysfunction and identify MYSM1 as a promising therapeutic target for reversing immunosuppression and preventing secondary infections in septic patients.

## Introduction

Sepsis is a life-threatening condition caused by a dysregulated host response to infection, which can lead to organ dysfunction and failure [1]. Clinically, sepsis is a major threat to the lives of hospitalized patients and is the leading cause of mortality in the intensive care unit (ICU) [2]. Most sepsis-related deaths occur beyond the first week of ICU admission, with secondary infections significantly contributing to increased mortality [3]. These infections, predominantly hospital-acquired and often antibiotic-resistant, challenge current treatment regimens [4–6].

The immune response in sepsis patients typically progresses from hyperactivation to suppression, with the latter stage predisposing patients to opportunistic infections [7]. Most clinical trials aimed at treating sepsis have focused on early-stage excessive inflammation and anti-inflammatory mechanisms [8–14]. Only a few studies have been designed to address later-stage immune suppression or to improve immune function [15–17]. However, to date, neither strategy has yielded new therapies that significantly improve the prognosis of sepsis patients.

Although previous reports have identified the role of adaptive immune changes, such as T-cell exhaustion, increased regulatory T cells (Tregs), and impaired B-cell function, in sepsis-induced immune suppression, relatively less research has been conducted on innate immunity and its role in sepsis-induced immune suppression [18]. Infection-induced epigenetic changes play a key role in the reprogramming of innate immune cells during long-term immune suppression in sepsis. Here, we report a disruption in deubiquitination, an epigenetic modification, that leads to the functional impairment of sepsis-associated innate immune cells, providing a critical foundation for sepsis-induced immunosuppression and secondary infections. More importantly, we identify MYSM1 as a key mediator in the immune response to sepsis, and targeting MYSM1 may offer a novel therapeutic strategy for managing sepsis-associated immune dysfunction.

## Results

### Immunosuppression in sepsis is associated with the dysfunction of immune cells

**Fig 1:**
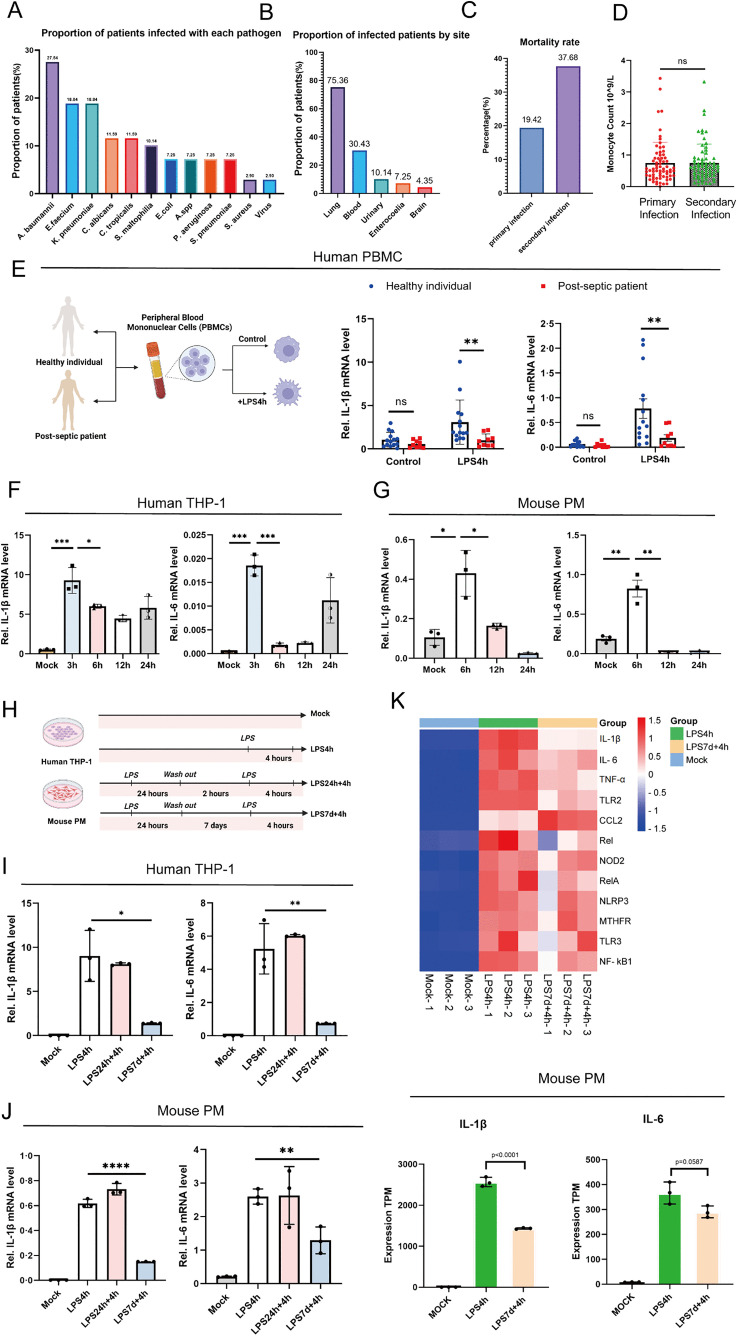
Immunosuppression in sepsis is associated with the dysfunction of immune cells. **(A)** Proportion of patients infected with each pathogen. Patients with polymicrobial infections are counted in each relevant category. The denominator is the total number of patients with secondary infections (n = 69). **(B)** Proportion of infected patients by site; Patients with multiple sites of secondary infection were counted in each relevant category. The denominator is the total number of patients with secondary infections (n = 69). **(C)** Mortality rates of sepsis patients with primary (n = 417) and secondary infections (n = 69). **(D)** Comparison of monocyte counts in sepsis patients with primary and secondary infections. **(E)** mRNA expression levels of IL-6 and IL-1β measured by RT-PCR in PBMCs isolated from healthy individuals (n = 14) and sepsis survivors (n = 9), with or without LPS stimulation for 4 hours; Fig 1E: Citation to Use: Created in BioRender. An, Q. (2026) https://BioRender.com/swt26rj. **(F)** THP-1 cells (a human acute monocytic leukemia cell line) were differentiated into macrophages by stimulation with 12-O-tetradecanoylphorbol-13-acetate (TPA), followed by LPS stimulation for 3, 6, 12, and 24 hours, and IL-6 and IL-1β mRNA expression levels were measured by RT-PCR. **(G)** Peritoneal macrophages (PMs) were isolated from wild-type (WT) C57BL/6 mice, followed by LPS stimulation for 6, 12, and 24 hours, and IL-6 and IL-1β mRNA expression levels were measured by RT-PCR. **(H)** A cell model of secondary infection induced by LPS in sepsis; Fig 1H: Citation to Use: Created in BioRender. An, Q. (2026) https://BioRender.com/tfpzsz9. **(I, J)** Stimulation methods of the LPS-induced secondary infection cell model applied to THP-1 and PMs, with IL-6 and IL-1β mRNA expression levels measured by RT-PCR. **(K)** PMs treated with stimulation methods from the LPS-induced secondary infection cell model, followed by transcriptomic sequencing to generate a heatmap of inflammatory gene expression. All experiments were repeated at least 3 times with similar results. The data are expressed as means ± SDs. Statistical analyses were performed using two-way ANOVA followed by Tukey’s multiple comparisons test **(E)**, and one-way ANOVA for panels (F, G, I, J, K). Data were considered statistically significant when *p ≤ 0.05, **p ≤ 0.01, ***p ≤ 0.001, and ****p ≤ 0.0001. https://doi.org/10.6084/m9.figshare.30581252.

We collected and analyzed clinical data from 486 sepsis patients and found that approximately 14.20% of them experienced secondary infections. The most common opportunistic pathogens involved in secondary infections included Acinetobacter baumannii, Klebsiella pneumoniae, and Enterococcus faecalis, with the majority being Gram-negative bacteria (Fig 1A). The main infection sites were the lungs and bloodstream (Fig 1B). Notably, the mortality rate associated with secondary infections was higher compared to primary infections (Fig 1C and S4 Table). Comparative analysis of the immune characteristics between primary and secondary infections in septic patients revealed no significant differences in peripheral blood immune cell counts (including leukocytes, neutrophils, lymphocytes, and monocytes), humoral immune markers, or cellular immune markers between the two groups (Figs 1D and S1). IL-6 and IL-1β, as key innate factors in host antimicrobial defense, play crucial roles in the immune response during sepsis [19,20]. We isolated peripheral blood mononuclear cells (PBMCs) from late-stage sepsis patients and healthy controls, followed by 4-hour LPS stimulation. Late-stage sepsis patients were defined as individuals meeting the Sepsis-3 criteria with a disease course of ≥7 days from diagnosis or ICU admission, who survived the initial septic insult but entered a prolonged immunosuppressive phase, characterized by persistent organ dysfunction, lymphopenia, and an increased risk or occurrence of secondary nosocomial infections. LPS-stimulated PBMCs from late-stage sepsis patients exhibited significantly reduced IL-6 and IL-1β levels compared to healthy controls (Fig 1E).

Furthermore, we conducted a time-course stimulation with LPS in human-derived THP-1 cells, mouse-derived PMs, and mouse BMDMs. The results showed that inflammatory cytokine expression peaked at 3–6 hours after LPS exposure (Figs 1F, 1G, S2A and S2B). Based on these findings, we established a secondary infection model in sepsis using these cells (Fig 1H), and observed that re-stimulation with LPS resulted in a blunted IL-6 and IL-1β response (Figs 1I, 1J, S2C and S2D). This was further supported by transcriptomic data showing the downregulation of inflammatory cytokine genes (Fig 1K).

These findings indicate that sepsis-induced immunosuppression primarily results from innate immune cell dysfunction rather than quantitative cellular deficiencies.

### H2AK119ub plays a critical role in immunosuppression during secondary infections in sepsis

Previous studies have shown that epigenetic modifications play a key role in the progression of sepsis [21]. Histone modifications induce immune cell reprogramming and contribute to the prolonged immunosuppressive state in sepsis [18,22]. Various types of histone modifications, including methylation [23,24], acetylation [25–28], ubiquitination [29,30], lactylation [31,32], and phosphorylation [33,34], play crucial roles in immune remodeling during different stages of sepsis.

**Fig 2:**
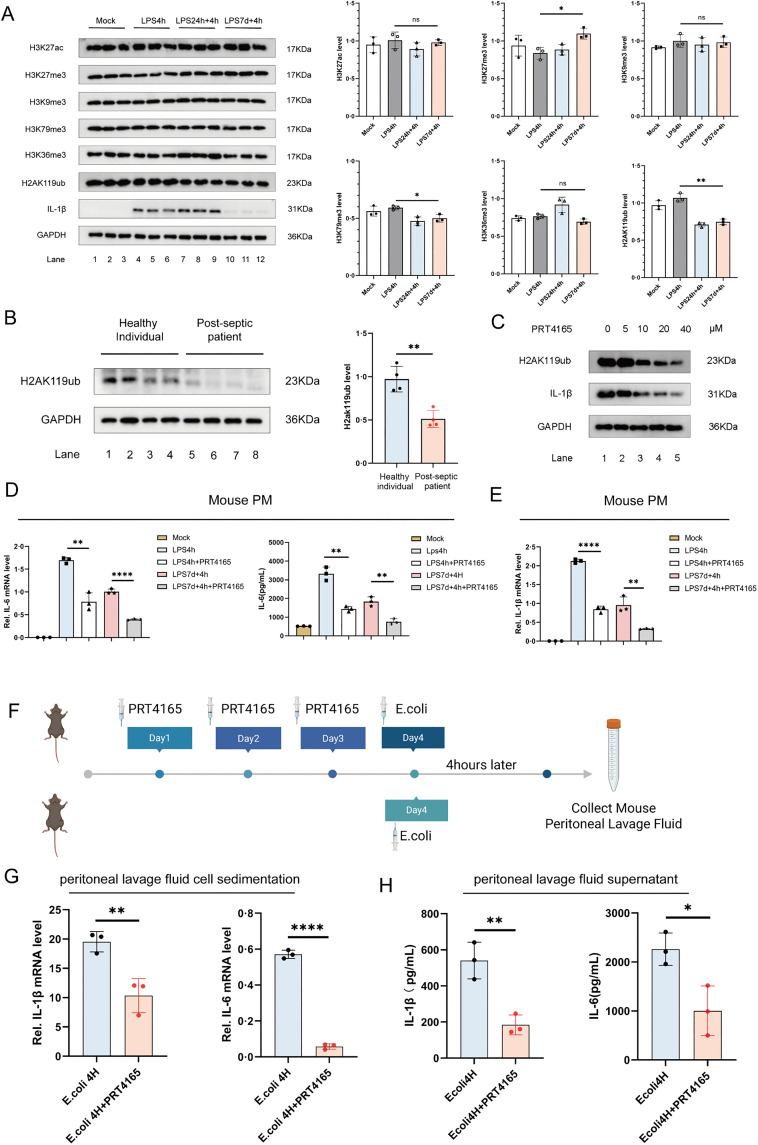
H2AK119ub plays a critical role in immunosuppression during secondary infections in sepsis. **(A)** Stimulation methods of the LPS-induced secondary infection cell model were applied to PMs derived from WT mice. Western blotting (WB) analysis was used to detect H3K27ac, H3K27me3, H3K9me3, H3K79me3, H3K36me3, and H2AK119ub levels. **(B)** WB analysis of H2AK119ub levels in peripheral blood PBMCs from healthy individuals and sepsis survivors. **(C)** PMs derived from WT mice were treated with different concentrations of the histone H2A ubiquitination inhibitor PRT4165 for 4 hours, followed by LPS stimulation for 4 hours. WB analysis was used to detect H2AK119ub and IL-1β levels. **(D, E)** Before the establishment of the LPS-induced secondary infection model in PMs from WT mice, PMs were treated with 10 μM PRT4165 for 4 hours. IL-6 and IL-1β mRNA expression levels were measured by RT-PCR, and the level of IL-6 protein in the cell supernatant was measured by ELISA. **(F)** Schematic representation of intraperitoneal injection of PRT4165 in WT mice. The experimental group (n=3) received intraperitoneal injections of PRT4165 (20 mg/kg) for 3 consecutive days, followed by intraperitoneal injection of an E. coli suspension (3 × 10⁸ cfu/mL, 1 mL/mouse) dissolved in PBS. The control group (n=3) received intraperitoneal injection of the E. coli suspension only. Peritoneal lavage fluid was collected 4 hours later. Fig 2F: Citation to Use: Created in BioRender. An, Q. (2026) https://BioRender.com/ss4krtx. **(G, H)** The collected peritoneal lavage fluid was centrifuged, and the cell pellet was analyzed for IL-1β and IL-6 mRNA expression levels using RT-PCR. The supernatant was analyzed for IL-1β and IL-6 protein levels using ELISA. All experiments were repeated at least 3 times with similar results. The data are expressed as means ± SDs. Statistical analysis was performed using two-sided Student’s t-test (B, G, H) and one-way ANOVA (A, D, E). Data were considered statistically significant when *p ≤ 0.05, **p ≤ 0.01, ***p ≤ 0.001, and ****p ≤ 0.0001.https://doi.org/10.6084/m9.figshare.30581426.

Analysis of acetylation, methylation, and ubiquitination-related proteins in primary and secondary sepsis models revealed elevated H3K27me3 and reduced H3K79me3 and H2AK119ub levels in the secondary LPS stimulation group compared to the primary group (Fig 2A). While the mechanisms of H3K79me3 and H3K27me3 methylation modifications in sepsis have been documented in the literature [35,36], the relationship between H2AK119ub and sepsis-induced immune suppression remains to be explored.

To investigate this further, we analyzed the levels of H2AK119ub in PBMCs from healthy individuals and sepsis patients in the later stage. Notably, H2AK119ub levels were significantly lower in late-stage sepsis patients than in healthy controls (Fig 2B). To further explore the relationship between H2AK119ub and sepsis-induced immunosuppression, we used PRT4165, a small-molecule inhibitor of the E3 ubiquitin ligase Ring1A/B, which effectively suppresses histone H2A lysine 119 ubiquitination [37]. We first established a concentration gradient for PRT4165 to determine the optimal dose for inhibiting H2AK119ub levels. The results showed that PRT4165 concentrations above 10 μM significantly inhibited H2AK119ub levels and also led to a marked reduction in IL-1β and IL-6 inflammatory cytokine levels (Figs 2C, S3A and S3B).

Building on this, we applied PRT4165 to both primary and secondary infection models of sepsis. The results revealed that PRT4165 attenuated primary LPS-induced inflammation and further diminished IL-6 and IL-1β levels in secondary stimulation (Fig 2D and 2E), highlighting H2AK119ub’s regulatory role in sepsis immunosuppression and cytokine expression. Next, we further validated the relationship between H2AK119ub and inflammatory levels in a mouse model (Fig 2F). Mice were treated with PRT4165 for three consecutive days to ensure an effective drug concentration. After pre-treatment with PRT4165, we injected the mice with a mixture of Escherichia coli on the fourth day to simulate bacterial infection and trigger an immune response. Four hours post-infection, we collected peritoneal lavage fluid for analysis. The results showed that PRT4165 pretreatment suppressed IL-6 and IL-1β levels in peritoneal lavage fluid following Escherichia coli infection (Fig 2G and 2H), reinforcing the link between H2AK119ub reduction and immunosuppression.

Therefore, these results underscore H2AK119ub’s critical role in sepsis-induced immunosuppression and its impact on inflammatory cytokine expression during secondary infections.

### MYSM1 interacts with H2AK119ub and reduces the level of H2AK119ub at the IL-6 and IL-1β promoters

To elucidate the mechanism by which H2AK119ub regulates sepsis-induced immunosuppression, we investigated factors influencing H2AK119ub levels. Previous studies have identified that ubiquitin-specific proteases, including USP16 (Ubp-M) [38], USP21 [39], USP22 [40], PR-DUB [41], and MYSM1 [42] are involved in the regulation of histone H2A. MYSM1 is a histone H2A deubiquitinase that specifically removes monoubiquitination at lysine 119, serves as a critical regulator of chromatin remodeling, transcriptional activation, hematopoiesis, and immune homeostasis, with its dysfunction leading to hematologic and immunologic disorders [43].

**Fig 3:**
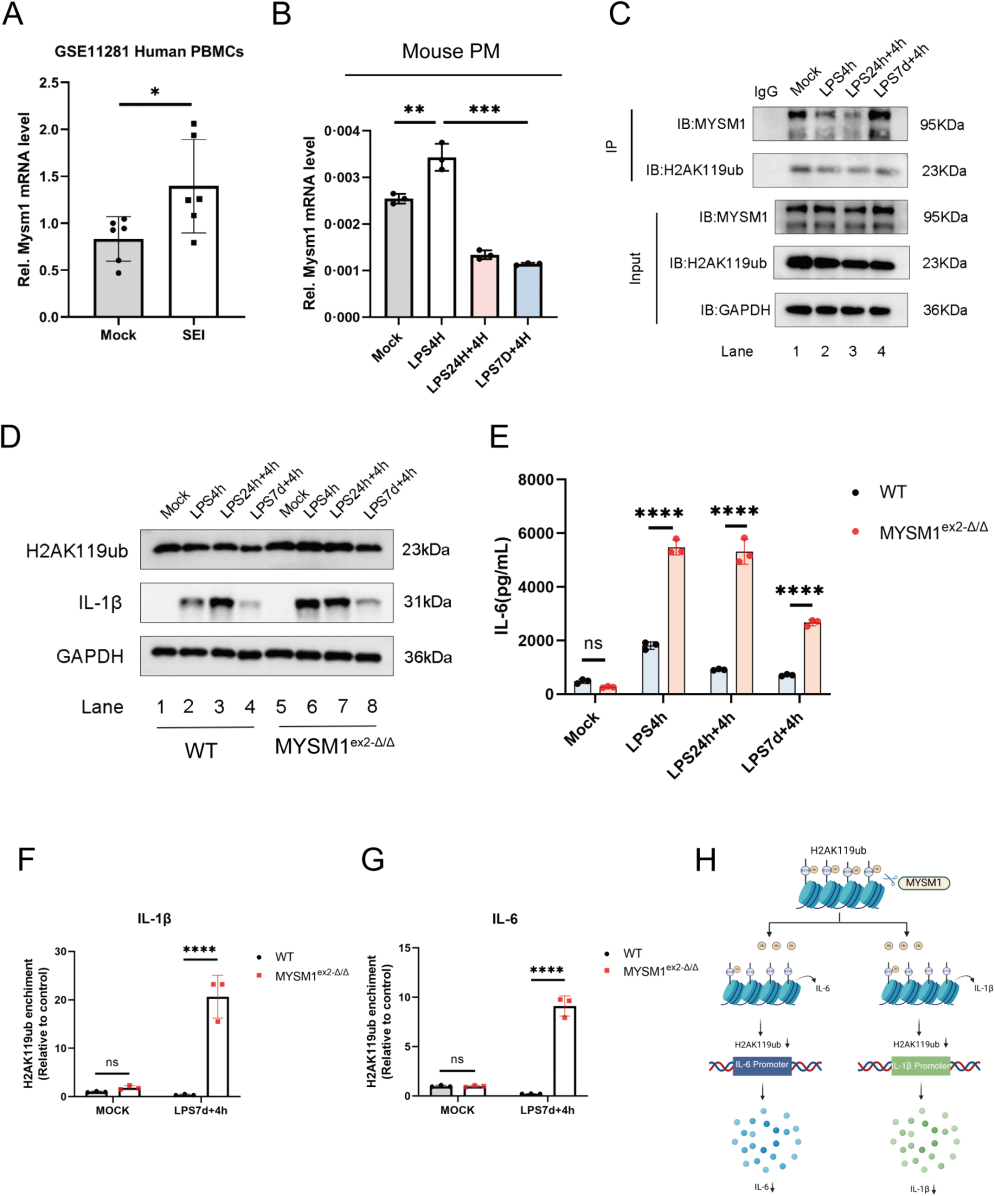
MYSM1 interacts with H2AK119ub and reduces the level of H2AK119ub at the IL-6 and IL-1β promoters. (A) MYSM1 expression in PBMCs from control and experimental groups exposed to Staphylococcus aureus SEI antigen for 6 hours, obtained from the GSE11281 dataset; (B) In the LPS-induced secondary infection cell model of sepsis using PMs derived from WT mice, MYSM1 mRNA expression levels were measured by RT-PCR. (C) In the LPS-induced secondary infection cell model of sepsis using PMs derived from WT mice, immunoprecipitation (Co-IP) and western blot (WB) analysis were performed to detect the interaction between H2AK119ub and MYSM1. (D, E) In the LPS-induced secondary infection cell model of sepsis using PMs derived from WT and MYSM1^ex2-Δ/Δ^ mice, H2AK119ub and IL-1β levels were detected by WB, IL-6 protein in the cell supernatant was measured by ELISA. (F, G) Cut&Tag-qPCR analysis of H2AK119ub enrichment at the IL-6 and IL-1β promoter regions in PMs derived from WT and MYSM1^ex2-Δ/Δ^ mice in an LPS-induced secondary infection sepsis model. (H) Mechanistic diagram illustrating that MYSM1 removes H2AK119 ubiquitination at the IL-6 and IL-1β promoter regions through its deubiquitinase activity, thereby suppressing the expression of the inflammatory cytokine IL-6 and IL-1β. Fig 3H: Citation to Use: Created in BioRender. An, Q. (2026) https://BioRender.com/eqf1660. All experiments were repeated at least 3 times with similar results. The data are expressed as means ± SDs. Statistical analysis was performed using two-way ANOVA followed by Tukey’s multiple comparisons test (E, F, G), two-sided Student’s t-test (A), and one-way ANOVA (B). Data were considered statistically significant when *p ≤ 0.05, **p ≤ 0.01, ***p ≤ 0.001, and ****p ≤ 0.0001. https://doi.org/10.6084/m9.figshare.30581489.

Analysis of the GSE11281 database revealed that Staphylococcus aureus SEI antigen significantly upregulated MYSM1 expression in PBMCs after 6 hours (Fig 3A). In primary and secondary sepsis models, MYSM1 expression increased significantly 4 hours after initial LPS stimulation (Fig 3B), indicating its responsiveness to bacterial antigens and immune stimuli. Subsequently, Co-immunoprecipitation experiments revealed that MYSM1 exhibited a stronger interaction with H2AK119ub during secondary LPS stimulation (Fig 3C).

To further investigate the role of MYSM1 in sepsis, MYSM1^ex^^2^^-Δ/Δ^ mice were generated using CRISPR/Cas9 technology (S4 and S5A Figs). The MYSM1 gene encodes nine transcripts; exon 2 of the MYSM1–201 transcript (ENSMUST00000075872.3), which contains a 76-bp coding sequence, was selected as the target region for deletion. An in-frame ATG codon located upstream of exon 3 can serve as an alternative translation initiation site, thereby permitting translation of the downstream coding sequence while eliminating the N-terminal region encoded by exon 2. Deletion of exon 2 resulted in the expression of a truncated MYSM1 protein that is smaller than the wild-type MYSM1 protein, which was experimentally confirmed by Western blot analysis (S5B Fig). The truncated protein retains the SWIRM and MPN domains and preserves catalytic deubiquitinase activity. Importantly, this truncation does not disrupt TRAF6/NOD2-mediated cytoplasmic ubiquitination–dependent NF-κB signaling, but selectively impairs the epigenetic regulatory function of MYSM1 (S5C Fig).

In contrast to complete MYSM1 knockout mice, MYSM1^ex2^^-Δ/Δ^ mice exhibited no overt developmental abnormalities, morphological deformities, or severe immune defects (S5D Fig). Body weight was comparable between MYSM1^ex^^2^^-Δ/Δ^ mice and wild-type littermates (S5E Fig). Flow cytometric analysis further demonstrated no significant differences in immune cell numbers between the two genotypes (S6 Fig). By contrast, complete loss of MYSM1 results in profound defects in hematopoietic and immune development, leading to markedly reduced yield, viability, and functional comparability of bone marrow– or peritoneum-derived macrophages. These systemic abnormalities substantially confound the interpretation of macrophage-intrinsic inflammatory responses in vitro under primary and secondary LPS challenge conditions, highlighting the suitability of the MYSM1^ex^^2^^-^^Δ/Δ^ model for dissecting MYSM1-dependent epigenetic regulation during sepsis.

Peritoneal macrophages were isolated from WT and MYSM1^ex2-Δ/Δ^ mice to establish primary and secondary infection models of sepsis. Experimental results showed that under secondary LPS stimulation, PMs from MYSM1^ex2-Δ/Δ^ mice displayed significantly higher levels of H2AK119ub,IL-1β, and IL-6 compared with WT mice (Fig 3D and 3E), suggesting that MYSM1 plays a crucial regulatory role in controlling H2AK119ub levels during LPS-induced immunosuppression. Biochemical validation of ubiquitination further confirmed that the observed H2AK119ub signal was specific to ubiquitinated H2A (S5F Fig).

Using CUT&Tag-qPCR, we assessed H2AK119ub enrichment at the IL-1β and IL-6 promoter regions. Compared with WT controls, MYSM1^ex2-Δ/Δ^ mice showed increased H2AK119ub enrichment at these promoters (Fig 3F and 3G). This indicates that MYSM1, through its deubiquitinase activity, regulates H2AK119ub levels at the IL-1β and IL-6 promoters, thereby impairing transcription initiation complex assembly and suppressing IL-1β and IL-6 expression (Fig 3H). Collectively, these findings highlight the pivotal role of MYSM1 in modulating H2AK119ub and its impact on inflammatory gene expression during sepsis-induced immunosuppression.

### Deletion of the N-terminal domain of MYSM1 upregulates H2AK119ub, promoting the inflammatory response

**Fig 4:**
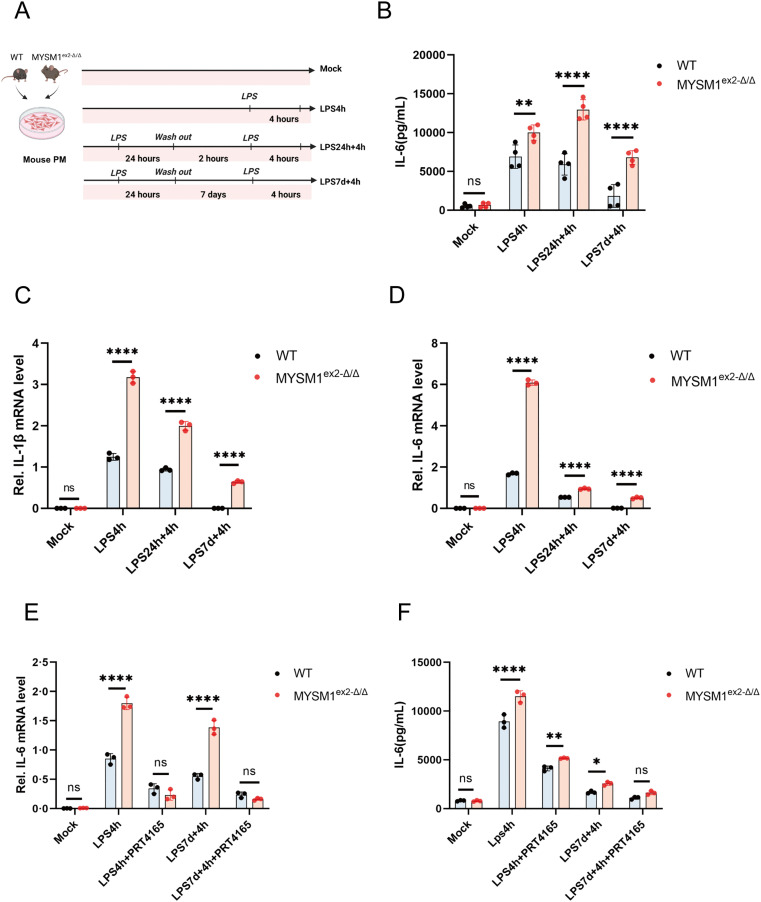
Deletion of the N-terminal domain of MYSM1 upregulates H2AK119ub, promoting the inflammatory response. (A) Schematic representation of the LPS-induced secondary infection cell model of sepsis using PMs derived from WT and MYSM1^ex2-Δ/Δ^ mice; Fig 4A: Citation to Use: Created in BioRender. An, Q. (2026) https://BioRender.com/lq1wp3y. (B, C, D) In the LPS-induced secondary infection model of sepsis using PMs derived from WT and MYSM1^ex2-Δ/Δ^ mice, IL-6 protein levels in the cell supernatant were detected by ELISA, and IL-6 and IL-1β mRNA expression levels in cell extracts were measured by RT-PCR. (E, F) Before inducing the secondary infection model of sepsis using PMs derived from WT and MYSM1^ex2-Δ/Δ^ mice with LPS, PMs were pretreated with 10 μM PRT4165 for 4 hours. The expression of IL-6 mRNA in the cell pellet was detected by RT-PCR, and the level of IL-6 protein in the cell supernatant was measured by ELISA. All experiments were repeated at least 3 times with similar results. The data are expressed as means ± SDs. Statistical analysis was performed using two-way ANOVA followed by Tukey’s multiple comparisons test (B, C, D, E, F). Data were considered statistically significant when *p ≤ 0.05, **p ≤ 0.01, ***p ≤ 0.001, and ****p ≤ 0.0001. https://doi.org/10.6084/m9.figshare.30581501.

To further investigate the regulatory role of MYSM1 on H2AK119ub, we isolated PMs from WT and MYSM1^ex^^2^^^-^Δ/Δ^ mice and established a secondary infection cell model (Fig 4A). After secondary LPS stimulation, we found that PMs from MYSM1^ex^^2^^-^^Δ/Δ^ mice exhibited significantly higher levels of IL-6 protein expression, as well as increased IL-6 and IL-1β mRNA expression, compared to WT mice(Fig 4B–D). These findings suggest that deletion of the N-terminal domain of MYSM1 promotes the expression of inflammatory cytokines and alleviates the immunosuppressive state in sepsis.

To confirm that the observed phenotypes are directly linked to MYSM1, rather than off-target CRISPR/Cas9 effects, we performed complementary gain- and loss-of-function experiments. Peritoneal macrophages isolated from MYSM1^ex^^2^^-Δ/Δ^ mice were transduced with AAV9 to overexpress MYSM1 (S7A Fig), followed by LPS stimulation to model secondary infection. Overexpression of MYSM1 in MYSM1^ex^^2^^-Δ/Δ^ mouse PMs via AAV9 markedly suppressed IL-1β and IL-6 mRNA expression upon secondary LPS stimulation, demonstrating a direct and cell-intrinsic role of MYSM1 in restraining IL-1β and IL-6 production and in maintaining endotoxin tolerance (S7B and S7C Fig). In parallel, MYSM1 was knocked down in THP-1 cells using siRNA (S7D Fig), and a similar LPS-induced secondary stimulation model was applied. Upon secondary LPS stimulation, IL-1β and IL-6 mRNA expression was markedly increased in MYSM1-deficient cells compared with controls, indicating that targeted MYSM1 knockdown partially enhances the immune response to secondary infection (S7E and S7F Fig). These results closely recapitulate the phenotype observed in PMs isolated from MYSM1^ex^^2^^-Δ/Δ^ mice, in which IL-1β and IL-6 mRNA expression was also significantly higher than in WT cells.

To further assess the involvement of H2AK119ub in this process, PMs were treated with PRT4165 prior to secondary LPS challenge. The results showed that there were no significant differences in IL-6 protein expression and IL-6 mRNA levels between MYSM1^ex^^2^^-Δ/Δ^ and WT mice PMs (Fig 4E and 4F). The use of PRT4165 can mimic the immunosuppressive state observed in secondary infections during sepsis. These experimental results further confirm the interaction between MYSM1 and H2AK119ub. Deletion of the N-terminal domain of MYSM1 upregulates H2AK119ub levels, thereby promoting the inflammatory response.

### Deletion of the N-terminal domain of MYSM1 enhances early inflammatory response in secondary infections in septic mice

**Fig 5:**
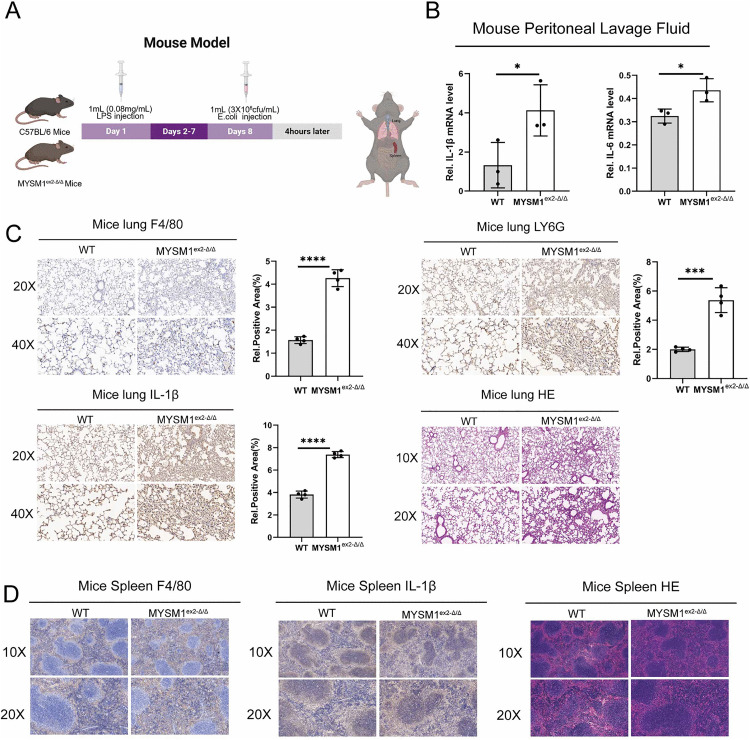
Deletion of the N-terminal domain of MYSM1 enhances early inflammatory response in secondary infections in septic mice. (A) According to the schematic diagram of the animal model 1, WT and MYSM1^ex2-Δ/Δ^ mice were intraperitoneally injected with LPS solution and E. coli suspension (3 × 10^8^ CFU/mL). Tissue collection and analysis were performed 4 hours post-injection; Fig 5A: Citation to Use: Created in BioRender. An, Q. (2026) https://BioRender.com/68gegzc. (B) Peritoneal lavage fluid was collected from WT (n = 3) and MYSM1^ex2-Δ/Δ^ mice (n = 3), and the mRNA expression levels of IL-1β and IL-6 were determined by RT-PCR. (C) Lung tissues were collected from WT (n = 4) and MYSM1^ex2-Δ/Δ^ mice (n = 4). The protein expression of F4/80, LY6G, and IL-1β in the lungs was examined by immunohistochemical analysis, and the histological features were evaluated using hematoxylin and eosin (H&E) staining. (D) Spleen tissues were collected from WT (n = 4) and MYSM1^ex2-Δ/Δ^ mice (n = 4), The protein expression of F4/80 and IL-1β in the spleen was examined by immunohistochemical analysis, and the histological morphology was analyzed using hematoxylin and eosin (H&E) staining. The data were expressed as means ± SDs. The statistical analysis was carried out using two-sided Student’s t-test (B, C). The data were considered statistically significant when *p ≤ 0.05, **p ≤ 0.01, ***p ≤ 0.001, and ****p ≤ 0.0001. https://doi.org/10.6084/m9.figshare.30581516.

Next, we further evaluated the impact of MYSM1 expression on sepsis-induced immune suppression in a mouse model (Fig 5A). On day 1, LPS was administered via intraperitoneal injection to WT and MYSM1^ex^^2^^-Δ/Δ^ mice. On day 8, the mice were infected with Escherichia coli to induce secondary stimulation, and tissues were collected 4 hours later (early secondary stimulation phase) for analysis. The results showed that MYSM1^ex^^2^^-Δ/Δ^ mice exhibited significantly higher IL-6 and IL-1β levels during the early secondary stimulation phase compared to WT mice (Fig 5B). Additionally, MYSM1^ex^^2^^-Δ/Δ^ mice exhibited heightened immune cell infiltration (macrophages and neutrophils) and inflammation in the lungs, accompanied by more severe tissue damage (Fig 5C). However, no significant differences were observed in immune cell infiltration, inflammation, or tissue damage in the spleen between WT and MYSM1^ex^^2^^-Δ/Δ^ mice at this time point (Fig 5D). These findings suggest that, in the early secondary stimulation phase, deletion of the N-terminal domain of MYSM1 induces a more robust inflammatory response.

### Deletion of the N-terminal domain of MYSM1 improves late survival and prognosis after secondary infection in septic mice

**Fig 6:**
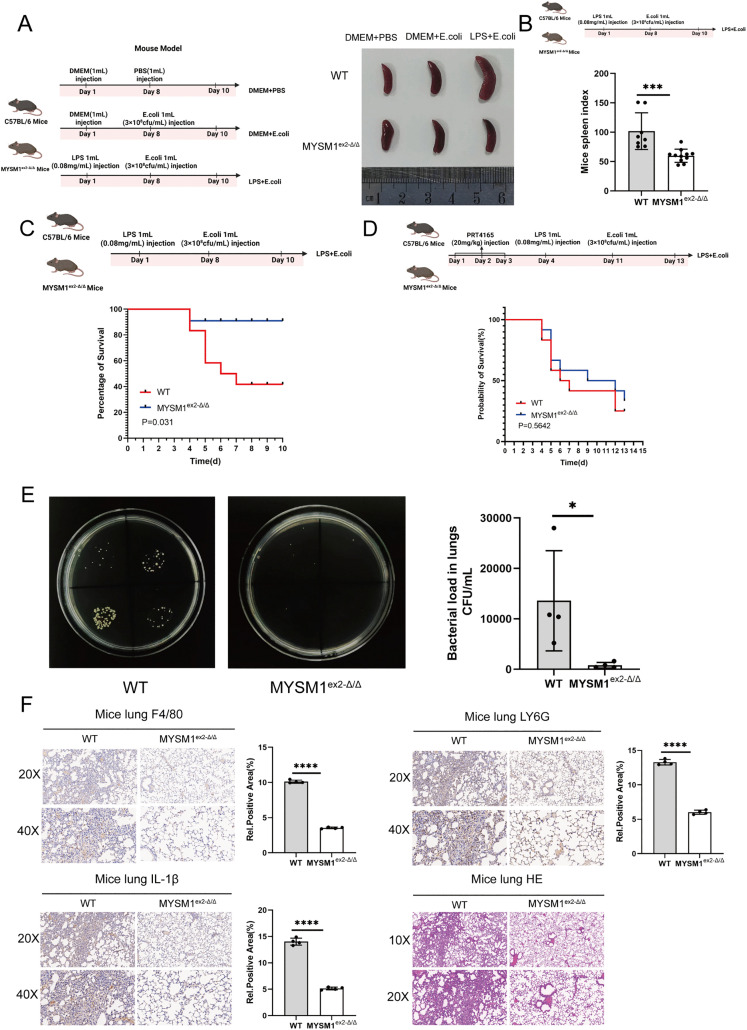
Deletion of the N-terminal domain of MYSM1 improves late survival and prognosis after secondary infection in septic mice. (A) Experimental mice were intraperitoneally injected according to the schematic diagram of the animal model 2. Subsequently, the spleens were isolated, and morphological observations were conducted and photographed; Fig 6A: Citation to Use: Created in BioRender. An, Q. (2026) https://BioRender.com/z5mdalr. (B) Spleens were isolated and weighed from the LPS pretreatment + *E. coli* infection group of WT (n = 8) and MYSM1^ex2-Δ/Δ^ (n = 11) mice, and the spleen index was analyzed for WT and MYSM1^ex2-Δ/Δ^ mice; Fig 6B: Citation to Use: Created in BioRender. An, Q. (2026) https://BioRender.com/4kjxje4. (C) Survival curves were recorded and analyzed for the LPS pretreatment + *E. coli* infection group of WT (n = 12) and MYSM1^ex2-Δ/Δ^ (n = 12) mice; Fig 6C: Citation to Use: Created in BioRender. Xjl, X. (2026) https://BioRender.com/okvc1xv. (D) Survival curve of WT (n = 12) and MYSM1^ex2-Δ/Δ^ (n = 12) mice pretreated with PRT4165 (20 mg/kg, once daily for 3 consecutive days) prior to intraperitoneal challenge with LPS or E. coli. Fig 6D: Citation to Use: Created in BioRender. An, Q. (2026) https://BioRender.com/kxjpyt0. (E) Bacterial load in the lung tissues was measured for the LPS pretreatment + *E. coli* infection group of WT (n = 4) and MYSM1^ex2-Δ/Δ^ (n = 4) mice. (F) Protein levels of F4/80, LY6G, and IL-1β in the lungs of the LPS pretreatment + *E. coli* infection group of WT (n = 4) and MYSM1^ex2-Δ/Δ^ (n = 4) mice were determined by immunohistochemical analysis. Histological features of the lung tissues were analyzed using hematoxylin and eosin (H&E) staining. The data were expressed as means ± SDs. Statistical analysis was performed using two-sided Student’s t-test (B, E, F) or Kaplan–Meier survival analysis (C, D). The data were considered statistically significant when *p ≤ 0.05, **p ≤ 0.01, ***p ≤ 0.001, and ****p ≤ 0.0001. https://doi.org/10.6084/m9.figshare.30581531.

To further investigate MYSM1’s role in the late phase of secondary infection, we extended E. coli stimulation to 48 h. Morphological analysis revealed that the spleens of MYSM1^ex^^2^^-Δ/Δ^ mice were significantly smaller and had a lower spleen index compared with WT controls following LPS and E. coli injection (Fig 6A and 6B). In addition, MYSM1^ex^^2^^-Δ/Δ^ mice showed reduced bacterial loads in lung tissues, diminished infiltration of macrophages and neutrophils, lower inflammatory marker expression, and markedly alleviated tissue damage during the late phase of secondary infection (Fig 6E and 6F). Consistently, survival analysis demonstrated that MYSM1^ex^^2^^-Δ/Δ^ mice had higher survival rates than WT mice (Fig 6C). However, when mice were pretreated with PRT4165 for three consecutive days before LPS or E. coli challenge, no significant differences in survival were observed between WT and MYSM1^ex^^2^^-Δ/Δ^ mice (Fig 6D). Collectively, these findings highlight the critical role of MYSM1-mediated epigenetic dysregulation in sepsis-induced immunosuppression. Deletion of the N-terminal domain of MYSM1 enhances the early inflammatory response and bacterial clearance, while in the late phase it mitigates tissue damage and improves survival outcomes.

## Discussion

This study identifies MYSM1 as a key deubiquitinase that mediates the immune response in sepsis by regulating histone ubiquitination status. Our results indicate that the dysfunction of innate immune cells is the primary cause of immunosuppression in sepsis. Furthermore, in contrast to the commonly studied histone modifications such as methylation and acetylation, which mediate immune remodeling in immune cells, we explored the role of ubiquitination modifications in macrophage immune dysfunction as a critical mechanism underlying sepsis-induced immunosuppression. Additionally, we found that late-stage sepsis-induced immunosuppression weakens early immune responses to bacterial stimulation, hindering the timely clearance of bacteria, resulting in delayed immune reactions that exacerbate tissue damage and contribute to sepsis-related mortality.

Previous studies have established MYSM1 as a critical negative regulator of innate immune signaling, functioning to restrain excessive inflammation and limit tissue damage. Mechanistically, MYSM1 interacts with TRAF3-, TRAF6-, and RIP2-containing signaling complexes to remove non-degradative K63-, K27-, and M1-linked polyubiquitin chains [44], thereby suppressing TLR- and NOD2-mediated inflammatory responses [45]. These immunoregulatory functions are primarily dependent on the coordinated action of the SWIRM and MPN/JAMM domains, whereas the N-terminal SANT domain is dispensable for cytoplasmic signaling control.

Structurally, MYSM1 is a modular deubiquitinase composed of an N-terminal SANT domain, a central SWIRM domain, and a C-terminal JAMM-type catalytic domain [43]. The SANT domain mediates chromatin anchoring and nuclear localization, enabling MYSM1 to associate with histones and regulatory genomic regions, while the SWIRM domain functions as a protein–protein interaction hub that facilitates assembly of transcriptional regulatory complexes in the nucleus and bridges immune signaling substrates in the cytoplasm. The MPN/JAMM domain constitutes the enzymatic core of MYSM1, conferring specificity toward H2AK119ub in the nucleus and toward signaling-associated polyubiquitin chains in the cytoplasm. Our findings extend this framework by demonstrating that deletion of the N-terminal SANT domain enhances immune activation during sepsis-induced immunosuppression, suggesting that partial disruption of MYSM1’s nuclear regulatory function may relieve immune tolerance and promote early pathogen clearance. MYSM1 exerts stage-dependent and domain-specific regulatory roles during sepsis, acting as a protective anti-inflammatory factor during hyperinflammation while contributing to immunosuppression at later stages. Further delineation of the temporal and compartment-specific functions of individual MYSM1 domains may provide new insights into therapeutic strategies targeting immune dysregulation in sepsis.

Historically, H2AK119ub has been widely regarded as a repressive histone modification central to Polycomb-mediated gene silencing [46–48]. Early studies linked H2AK119ub accumulation to transcriptional repression of Hox genes and the inactive X chromosome [47], and mechanistic work showed that this mark can block H3K4 methylation, inhibit transcriptional elongation by preventing FACT recruitment [39,49], and promote H3K27me3 deposition through PRC2 recruitment [50]. These findings established H2AK119ub as an important mediator of transcriptional repression. However, subsequent research has revealed that H2AK119ub also exerts transcriptional activating functions in a context-dependent manner. For example, ZRF1 was shown to displace PRC1 from chromatin via H2AK119ub binding and facilitate transcriptional activation, and recent work demonstrated that PRC1 can promote gene expression by modulating higher-order chromatin architecture [51]. More strikingly, Li et al. provided direct evidence that H2AK119ub fine-tunes transcription bidirectionally: it can counteract cPRC1-mediated chromatin compaction while simultaneously enhancing H1-dependent compaction, thus exerting dual effects on gene expression [52]. Complementarily, Wu et al. demonstrated that ncPRC1.1-catalyzed H2AK119ub enriches at active promoters in regulatory T cells and licenses transcriptional responses essential for immune adaptation [53]. Consistent with these emerging insights, our study identifies a novel role of MYSM1 in regulating H2AK119ub occupancy at IL-6 and IL-1β promoter regions, thereby driving cytokine induction and immune responses during sepsis. These findings extend the growing body of evidence that H2AK119ub is not merely a repressive mark, but a versatile epigenetic regulator capable of exerting both silencing and activating functions depending on chromatin context and cellular state.

Despite highlighting the important role of MYSM1 in regulating immune responses during sepsis, several limitations of this study should be acknowledged. First, our investigation primarily focused on the regulation of IL-6 and IL-1β; however, MYSM1 may modulate immune responses through additional cytokines or immune mediators. Future studies incorporating a broader cytokine and inflammatory mediator profile will be necessary to comprehensively define the immunoregulatory network governed by MYSM1. Second, although we demonstrate that deletion of the N-terminal SANT domain enhances immune activation during late-stage sepsis, the precise molecular mechanisms by which this domain regulates epigenetic modifications remain incompletely defined. The distinct regulatory functions of individual MYSM1 domains warrant further in-depth investigation. Moreover, the therapeutic potential of targeting MYSM1 requires careful evaluation. Given the stage-dependent roles of MYSM1 during sepsis, therapeutic strategies aimed at modulating MYSM1 activity must be cautiously designed, as maintaining immune balance is critical for immune homeostasis. Future studies should focus on optimizing MYSM1-targeted interventions and developing stage-specific therapeutic strategies for sepsis.

In conclusion, this study demonstrates that MYSM1-mediated epigenetic dysregulation drives immunosuppression and secondary infections in sepsis. Our findings link histone ubiquitination to cytokine expression, offering insights into immune response regulation during sepsis. These results provide a theoretical foundation for MYSM1’s role in immune regulation and highlight its potential as a therapeutic target in sepsis.

## Conclusions

**Fig 7:**
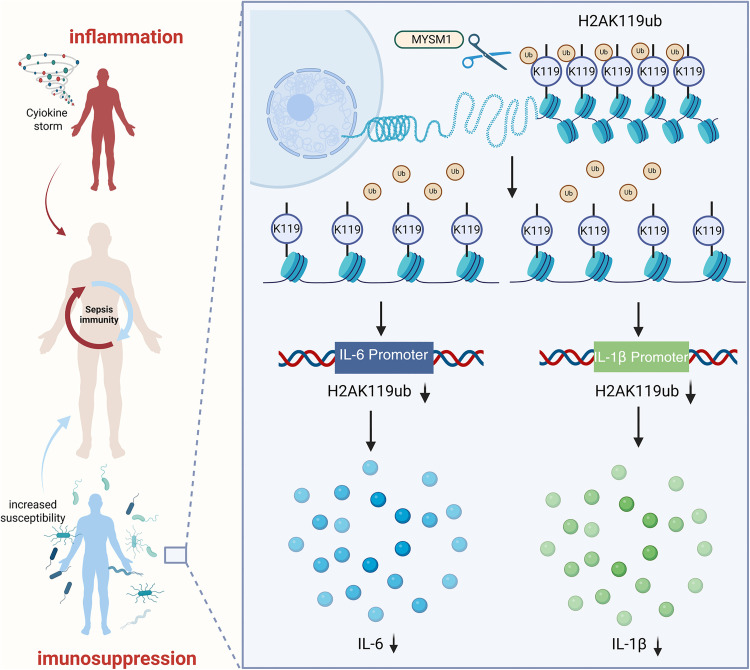
Model of MYSM1-mediated epigenetic regulation in sepsis. MYSM1 controls H2AK119ub deposition at IL-6 and IL-1β promoters, shaping immune dynamics during secondary infection. Fig 7: Citation to Use: Created in BioRender. An, Q. (2026) https://BioRender.com/ey2ee9y. https://doi.org/10.6084/m9.figshare.30581543.

Our study reveals that the late stage of sepsis is characterized by a state of immune suppression, closely associated with functional loss of immune cells. We identify H2AK119ub as a key epigenetic mark driving this immunosuppressive phenotype. Pharmacological inhibition of histone H2A ubiquitination by PRT4165 dampens immune responses to external stimuli, thereby mimicking the immunosuppressive state observed in sepsis. Mechanistically, MYSM1 regulates H2AK119ub at the promoters of IL-6 and IL-1β, playing a pivotal role in immune cell dysfunction. Targeted deletion of the N-terminal domain of MYSM1 reactivates early immune responses during secondary infection in septic mice, facilitating pathogen clearance and improving late-stage survival outcomes. These findings define MYSM1-mediated H2AK119ub regulation as a central epigenetic mechanism underlying sepsis-induced immune paralysis, offering potential therapeutic insight into restoring immune competence during sepsis (Fig 7).

### Methods

#### Ethics statement.

The study adhered to the Declaration of Helsinki and was approved by the Institutional Review Board (IRB) of Renmin Hospital of Wuhan University (Approval Number WDRY2024-K100). Blood sample collection followed guidelines for human subject protection, and written informed consent was obtained from all participants. Animal studies complied with the Animal Welfare Act and NIH guidelines for laboratory animal care and use, with protocols approved by the Institutional Animal Care and Use Committee (IACUC) of Renmin Hospital of Wuhan University (Approval Number WDRY IACUC 20240301A).

### Experimental model and subject details

#### Human PBMC analysis.

Blood samples from healthy individuals and sepsis recovery patients were collected from Renmin Hospital of Wuhan University. PBMCs were isolated by diluting blood in 5 mL RPMI-1640 medium (GIBCO, Grand Island, NY, USA) and layering it over 5 mL lymphocyte separation medium (#50494, MP Biomedicals, Santa Ana, CA, USA), followed by centrifugation at 2000 × g for 10 min. The middle layer was transferred, diluted with RPMI-1640, and red blood cells were lysed using a lysis buffer (Sigma-Aldrich, St. Louis, MO, USA). PBMCs were cultured in RPMI-1640 medium supplemented with 10% FBS, 100 U/mL penicillin, and 100 μg/mL streptomycin at 37°C in 5% CO_2_.

#### Animal study.

C57BL/6 WT mice were obtained from Hubei Research Center of Laboratory Animals, and MYSM1^−/−^ mice on a C57BL/6 background were provided by Dr. Xiaoxia Jiang (Academy of Military Medical Sciences, Beijing). Male mice were raised until 8 weeks old and used for infection or cell experiments. All procedures adhered to the Animal Welfare Act and NIH guidelines for the care and use of laboratory animals and were approved by the Institutional Animal Care and Use Committee (IACUC) of Renmin Hospital of Wuhan University.

#### Generation of MYSM1^ex2-Δ/Δ^ mice by CRISPR/Cas9 technology.

MYSM1^ex2-Δ/Δ^ mice were generated using CRISPR/Cas9 technology. The MYSM1 gene contains nine transcripts, among which exon 2 of the MYSM1–201 (ENSMUST00000075872.3) transcript, containing a 76 bp coding sequence, was selected as the target region for knockout. Deletion of this region leads to disruption of MYSM1 protein function. In this project, CRISPR/Cas9 technology was applied to modify the Mysm1 gene. Specifically, sgRNA was transcribed in vitro, and Cas9 mRNA together with sgRNA was microinjected into fertilized eggs of C57BL/6JGpt mice. The injected embryos were transplanted into pseudopregnant females to obtain F0 founder mice. Genetically modified F0 mice were identified by PCR amplification and sequencing. Positive F0 mice were then crossed with wild-type C57BL/6JGpt mice to obtain F1 offspring carrying the desired genetic modification. Homozygous MYSM1^ex2-Δ/Δ^ mice were obtained by intercrossing heterozygous F1 mice. Transgenic mice were generated and supplied by GemPharmatech Co., Ltd. (Nanjing, China). The primers used for genotyping are as S1 Table.

#### Cell lines and cultures.

The human monocytic cell line THP-1 (TIB-202) was obtained from ATCC (Manassas, VA, USA) and kindly provided by Dr. Bing Sun (Institute of Biochemistry and Cell Biology, Shanghai, China). THP-1 cells were maintained in RPMI-1640 medium (Gibco, Grand Island, NY, USA) supplemented with 10% fetal bovine serum (FBS), 100 U/mL penicillin, and 100 μg/mL streptomycin. Cells were cultured at 37°C in a humidified incubator containing 5% CO₂. The Escherichia coli DH5α strain (Cat. CD201, TRANSGEN Biotech, Beijing, China) was used for intraperitoneal injection in mouse models.

#### Reagents and antibodies.

Trizol reagent (15596018) was purchased from Ambion (Austin, TX, USA). Mouse IL-6 ELISA kit (431307) and Mouse IL-1β ELISA kit (432604) were purchased from BioLegend (San Diego, CA, USA). PRT4165 (T3110) was purchased from TargetMol (Boston, MA, USA). The following antibodies were used in this study: anti-MYSM1 (ab193081) and anti-F4/80 (ab300421) were purchased from Abcam (Cambridge, UK). Anti-H2AK119ub (8240S), anti-H3K36me3 (4909S), anti-H3K79me3 (4260S), anti-H3K9me3 (13969T), anti-H3K27me3 (9733T), anti-H3K27ac (8173T), and anti-H2A (12349S) were purchased from Cell Signaling Technology (Danvers, MA, USA). Anti-GAPDH (G9295) was purchased from Sigma-Aldrich (St. Louis, MO, USA). Anti-Rabbit IgG FITC (A22120), anti-Rabbit IgG CY3 (A22220), and anti-Mouse IgG CY3 (A22210) were purchased from Abbkine (Wuhan, China). Anti-Mouse IgG FITC (20000029) was purchased from Proteintech (Wuhan, China). Anti-IL-1β (AF-401-NA) was purchased from R&D Systems (Minneapolis, MN, USA). Anti-LY6G (GB11229) was purchased from Servicebio (Wuhan, China).

#### Cell model establishment.

PM and THP-1 cells were seeded in 12-well plates at a density of 6 × 10^5^ cells per well. Four experimental groups were established: (1) no stimulation; (2) stimulation with 5 μL/well LPS (100 μg/mL) for 4 hours; (3) stimulation with 5 μL/well LPS for 24 hours, followed by PBS washing, 2-hour rest, and re-stimulation with 5 μL/well LPS for 4 hours; (4) stimulation with 5 μL/well LPS for 24 hours, followed by PBS washing, 7-day rest, and re-stimulation with 5 μL/well LPS for 4 hours.

### Mouse model establishment

#### Animal model 1.

WT and MYSM1^ex^^2^^-Δ/Δ^ mice were intraperitoneally injected with 1 mL serum-free DMEM containing LPS (4 mg/kg) on day 1. On day 8, mice were injected with 1 mL *E. coli* suspension (3 × 10⁸ CFU/mL) in PBS. Samples were collected 4 hours post-injection.

#### Animal model 2.

WT and MYSM1^ex2-Δ/Δ^ mice were divided into three groups:

**Control group:** Mice were injected with 1 mL serum-free DMEM on day 1 and 1 mL PBS on day 8. Samples were collected 48 hours post-injection.

**E. coli infection group:** Mice were injected with 1 mL serum-free DMEM on day 1 and 1 mL *E. coli* suspension (3 × 10⁸ CFU/mL) on day 8. Samples were collected 48 hours post-injection.

**LPS pretreatment + E. coli infection group:** Mice were injected with 1 mL serum-free DMEM containing LPS (4 mg/kg) on day 1 and 1 mL *E. coli* suspension (3 × 10⁸ CFU/mL) on day 8. Samples were collected 48 hours post-injection.

### Method details

#### Isolation method for Peritoneal Macrophages (PMs).

Eight- to ten-week-old mice were euthanized using carbon dioxide (CO₂) inhalation, followed by sterilization with 75% ethanol. The peritoneum was exposed, and 6–8 mL of 1 × PBS was injected into the peritoneal cavity using a 10 mL syringe to collect peritoneal fluid. The fluid was centrifuged at 1500 rpm for 5 minutes, and the cell pellet was resuspended in DMEM supplemented with 10% FBS and 1% penicillin-streptomycin. Cells were cultured at 37°C in 5% CO₂. After 24 hours, the medium was replaced, and non-adherent cells were removed by PBS washing before further experiments.

#### RT-PCR and ELISA.

Total RNA was extracted from tissues or cells using Trizol reagent (Invitrogen). cDNA was synthesized from 1 µg RNA using reverse transcription mix (Vazyme Biotech). RT-PCR was performed with specific primers and ChamQ SYBR qPCR Master Mix (Vazyme Biotech). Cytokine levels in cell supernatant were measured using an ELISA kit, following the manufacturer’s instructions. Primer sequences were as S2 Table.

#### Immunoblotting.

Samples were diluted with 2 × loading buffer, boiled for 10 minutes, and separated by polyacrylamide gel electrophoresis. Proteins were transferred to a PVDF membrane and blocked with 5% non-fat milk in TBST (0.05% Tween 20) for 1 hour at room temperature. The membrane was washed three times with TBST and incubated overnight at 4°C with primary antibody diluted in TBST containing 5% BSA. After washing, the membrane was incubated with secondary antibody in 3% non-fat milk/TBST for 1 hour at room temperature. Protein bands were visualized using the ChemiDoc Imaging System (Bio-Rad) after final TBST washes.

#### Co-immunoprecipitation.

Cells were harvested using ice-cold 1 × PBS and lysed in 1000 μL lysis buffer (20 mM Tris-HCl, pH 7.4–7.5, 150 mM NaCl, 1 mM EDTA, 1% Nonidet P-40) containing phosphatase and protease inhibitors at 4°C for 1 hour. H2AK119ub and IgG antibodies were added to the lysate as positive and negative controls, respectively, and incubated with protein G agarose for 4 hours. Immunoprecipitates were washed three times with pre-lysis buffer, and target protein expression was analyzed by Western blot.

#### Histological analysis and immunohistochemistry.

Experiments were performed according to the literature. Mice's  different organs (spleen and lung) were fixed in 4% paraformaldehyde, embedded in paraffin, cut into sections, and placed on adhesion microscope slides. Sections were subjected to immunohistochemical (IHC) staining according to standard procedures.

#### Cut&Tag qPCR.

The Cut&Tag qPCR experiments were conducted using Hyperactive Universal CUT&Tag Assay Kit from Vazyme (TD904), and experiments were conducted according to the instructions. The qPCR primers were as S3 Table.

#### Genotype identification.

A 1–2 cm tail tissue sample was excised and lysed in a buffer containing Proteinase K (500 µL) and Proteinase K Buffer (20 µL) at 56°C for 12 hours. After centrifugation at 12,000 rpm for 5 minutes, 500 µL of supernatant was transferred to a new tube. DNA was precipitated with 500 µL isopropanol, inverted, and centrifuged at 12,000 rpm for 5 minutes. The pellet was washed with 75% ethanol, centrifuged at 12,000 rpm for 1 minute, and air-dried. DNA was resuspended in 20 µL ddH₂O. Target genes were amplified using specific PCR primers, and products were analyzed by 2% agarose gel electrophoresis to determine the genotype. The qPCR primers were as S1 Table.

#### Determination of lung tissue bacterial load.

Half of the lung tissue was harvested from experimental mice, cut into small pieces under sterile conditions, and homogenized in 500 µL PBS. The resulting suspension was diluted twofold, and 10 µL was plated onto agar plates divided into four sections. Plates were incubated for 24–48 hours, and bacterial colonies were counted to calculate colony-forming units (CFU/mL) in the tissue.

#### Statistical analysis.

Immunoblotting and histological analysis were quantified using ImageJ. Data from at least three independent experiments were expressed as means ± SDs. Statistical analysis was performed using GraphPad Prism (v9.5.1). Two-tailed Student’s t-test was used for comparisons between two groups, while one-way or two-way ANOVA followed by Tukey’s multiple comparison test was applied for multiple-group comparisons. A P-value <0.05 was considered statistically significant (*P < 0.05, **P < 0.01, ***P < 0.001, and ****P < 0.0001).

## Supporting information

S1 Fig Clinical data analyses. Related to Fig 1D. Immune cell counts and cytokine data in sepsis patients during primary and secondary infections. The data are expressed as means ± SDs. Statistical analysis was carried out using the t-test. The data were considered statistically significant when P ≤ 0.05 (*), P ≤ 0.01 (**), P ≤ 0.001 (***). https://doi.org/10.6084/m9.figshare.30581558.
(TIF)

S2 Fig LPS-induced immunosuppression in BMDMs. (A, B) Bone marrow–derived macrophages (BMDMs) were isolated from WT mice and stimulated with LPS for 6, 12, or 24 hours. IL-1β and IL-6 mRNA levels were quantified by RT-PCR; (C, D) To model secondary infection in sepsis, BMDMs were stimulated with LPS, and IL-1β and IL-6 mRNA expression was measured by RT-PCR. The data are expressed as means ± SDs. Statistical analysis was carried out using one-way ANOVA. The data were considered statistically significant when P ≤ 0.01 (**). https://doi.org/10.6084/m9.figshare.30581567.
(TIF)

S3 Fig Stimulation with a gradient of PRT4165. Related to Fig 2C. (A, B) WT peritoneal macrophages were treated with increasing concentrations of the histone H2A ubiquitination inhibitor PRT4165 for 4 h, followed by LPS stimulation for 4 h. IL-1β and IL-6 mRNA expression was quantified by RT-PCR. The data are expressed as means ± SDs. Statistical analysis was carried out using one-way ANOVA. The data were considered statistically significant when P ≤ 0.05 (*), P ≤ 0.01 (**). https://doi.org/10.6084/m9.figshare.30581573.
(TIF)

S4 Fig Gene Identification Diagram of MYSM1^ex^^2^^-Δ/Δ^ Mice. Genotyping was performed on tail tissues excised from WT and MYSM1^ex^^2^^-Δ/Δ^ mice. The qPCR primers were as S1 Table. https://doi.org/10.6084/m9.figshare.30581582.
(TIF)

S5 Fig Phenotypic characteristics of MYSM1^ex^^2^^-Δ/Δ^ mice. (A) Schematic diagram of the CRISPR/Cas9-based design strategy for generating MYSM1^ex^^2^^-Δ/Δ^ mice. S5A Fig: Citation to Use: Created in BioRender. An, Q. (2026) https://BioRender.com/7eganov. (B) MYSM1 protein levels in peritoneal macrophages from WT and MYSM1^ex^^2^^-Δ/Δ^ mice were detected by WB. (C) In the LPS-induced secondary infection cell model of sepsis using PMs derived from WT and MYSM1^ex^^2^^-Δ/Δ^ mice, P-P65 protein levels were detected by WB. (D) Representative gross appearance of WT and MYSM1^ex^^2^^-Δ/Δ^ mice. (E) Body weight of WT(n = 12) and MYSM1^ex^^2^^-Δ/Δ^ mice(n = 12). (F) In the LPS-induced secondary infection cell model of sepsis using PMs derived from WT and MYSM1^ex^^2^^-Δ/Δ^ mice, immunoprecipitation (Co-IP) and western blot (WB) analysis were performed to validate the specificity of the H2AK119ub antibody. https://doi.org/10.6084/m9.figshare.30581600.
(TIF)

S6 Fig Flow cytometric analysis of immune cells. Flow cytometry analysis of immune cell composition in the spleens or bone marrow from WT mice (n = 3) and MYSM1^ex^^2^^-Δ/Δ^ mice (n = 3). The data were expressed as means ± SDs. The statistical analysis was carried out using the t test. The data were considered not statistically significant when (p ＞ 0.05). https://doi.org/10.6084/m9.figshare.30581633.
(TIF)

S7 Fig MYSM1 gain- and loss-of-function experiments. (A) Peritoneal macrophages were isolated from WT and MYSM1^ex^^2^^-Δ/Δ^ mice, transduced with AAV9 to overexpress MYSM1, and MYSM1 mRNA expression was assessed by RT-PCR. (B, C) Peritoneal macrophages isolated from MYSM1^ex^^2^^-Δ/Δ^ mice were transduced with AAV9 to overexpress MYSM1, followed by LPS stimulation to model secondary infection. The transcriptional levels of IL-1β and IL-6 were then assessed by RT-PCR. (D) MYSM1 was knocked down in THP-1 cells using siRNA, and MYSM1 mRNA expression was assessed by RT-PCR. (E, F) MYSM1 was knocked down in THP-1 cells using siRNA, followed by LPS stimulation to model secondary infection, and IL-1β and IL-6 mRNA levels were measured by RT-PCR. https://doi.org/10.6084/m9.figshare.31101058.
(TIF)

S1 Table The primer sequences used for genotyping. https://doi.org/10.6084/m9.figshare.31127227.
(XLSX)

S2 Table RT-PCR Primer sequences. https://doi.org/10.6084/m9.figshare.31127245.
(XLSX)

S3 Table Cut&Tag qPCR primer sequences. https://doi.org/10.6084/m9.figshare.31127251.
(XLSX)

S4 Table Comparison of Clinical Characteristics Between Primary and Secondary Infection Data Sets in Sepsis. https://doi.org/10.6084/m9.figshare.31127254.
(XLSX)

S1 Data The values used to build graphs in this study. https://doi.org/10.6084/m9.figshare.30581651.
(XLSX)

S2 Data Clinical Characteristics of Patients with Primary and Secondary Infections. https://doi.org/10.6084/m9.figshare.30581657. (XLSX)

S3 Data Raw Image File from Western Blots. https://doi.org/10.6084/m9.figshare.30581675.
(DOCX)
