# Supplementary figures and images for: MYSM1-mediated epigenetic modification dysregulation leads to immunosuppression and secondary infections in sepsis
Source: PLoS Pathog. 2026 Feb 12;22(2):e1013935. doi: 10.1371/journal.ppat.1013935 (PMC12928581; doi:10.1371/journal.ppat.1013935)

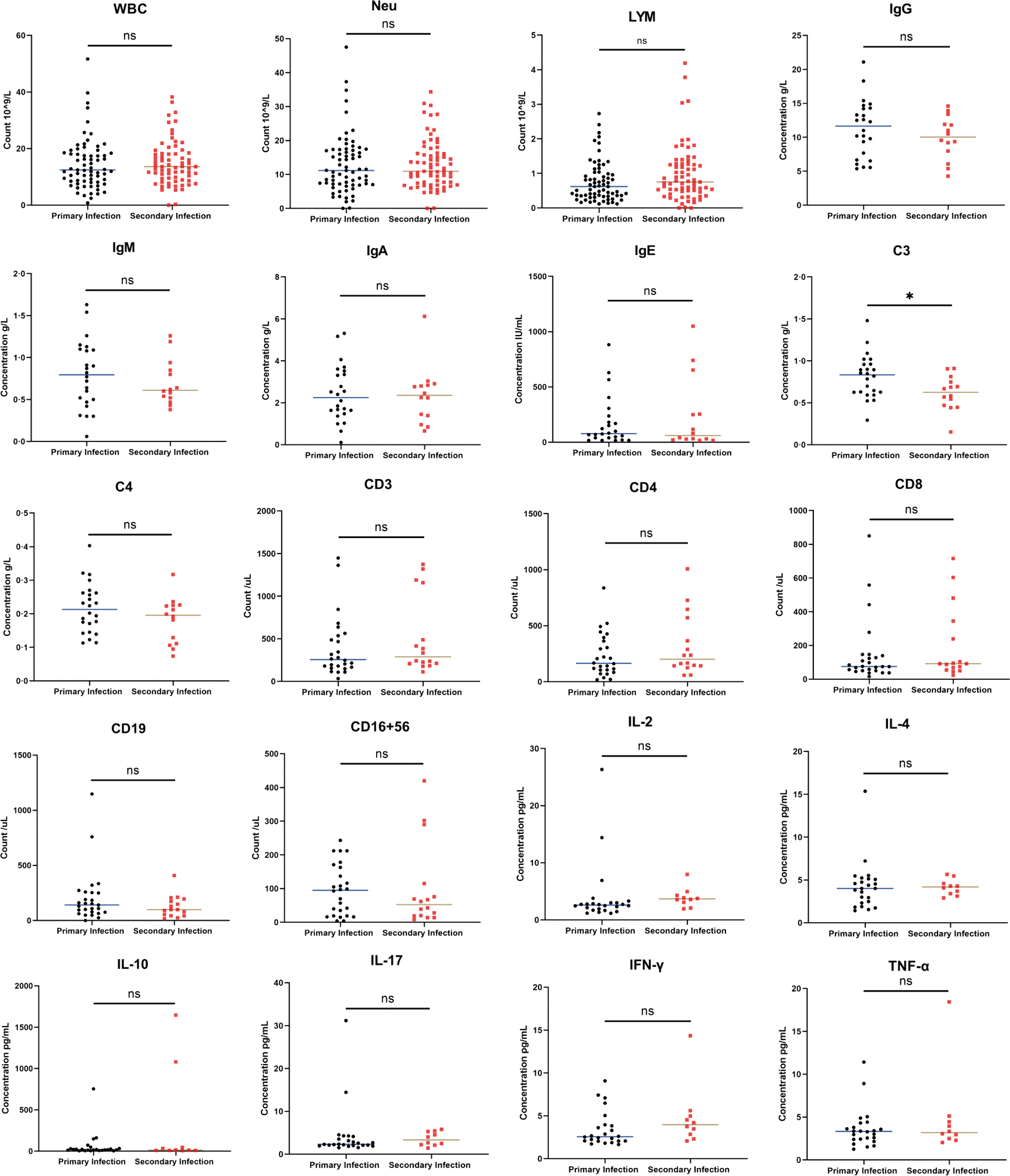

Supplement: S1 Fig — Related to Fig 1D. Immune cell counts and cytokine data in sepsis patients during primary and secondary infections. The data are expressed as means ± SDs. Statistical analysis was carried out using the t-test. The data were considered statistically significant when P ≤ 0.05 (*), P ≤ 0.01 (**), P ≤ 0.001 (***). https://doi.org/10.6084/m9.figshare.30581558. (TIF) [file ppat.1013935.s001.tif]

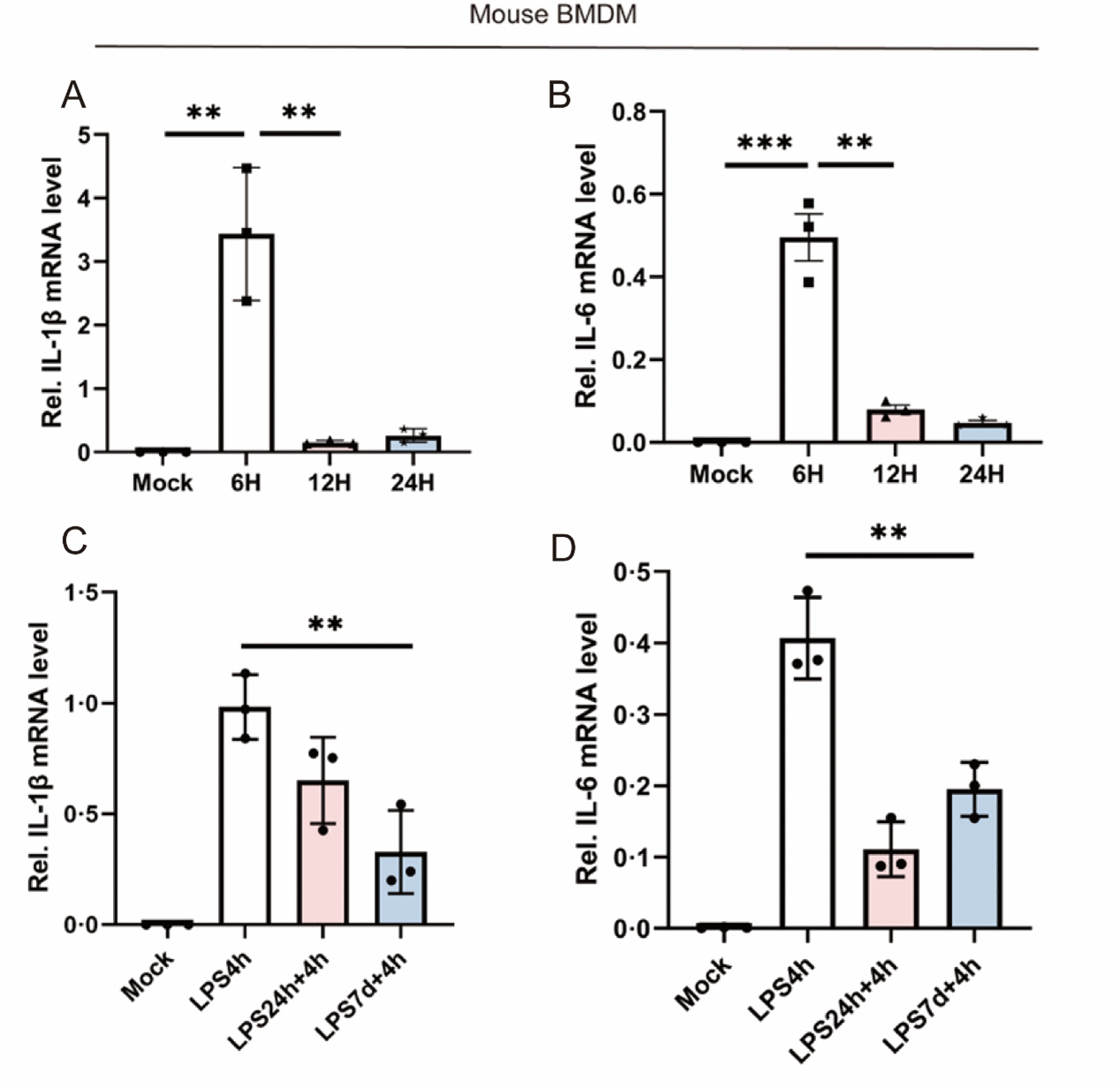

Supplement: S2 Fig — (A, B) Bone marrow–derived macrophages (BMDMs) were isolated from WT mice and stimulated with LPS for 6, 12, or 24 hours. IL-1β and IL-6 mRNA levels were quantified by RT-PCR; (C, D) To model secondary infection in sepsis, BMDMs were stimulated with LPS, and IL-1β and IL-6 mRNA expression was measured by RT-PCR. The data are expressed as means ± SDs. Statistical analysis was carried out using one-way ANOVA. The data were considered statistically significant when P ≤ 0.01 (**). https://doi.org/10.6084/m9.figshare.30581567. (TIF) [file ppat.1013935.s002.tif]

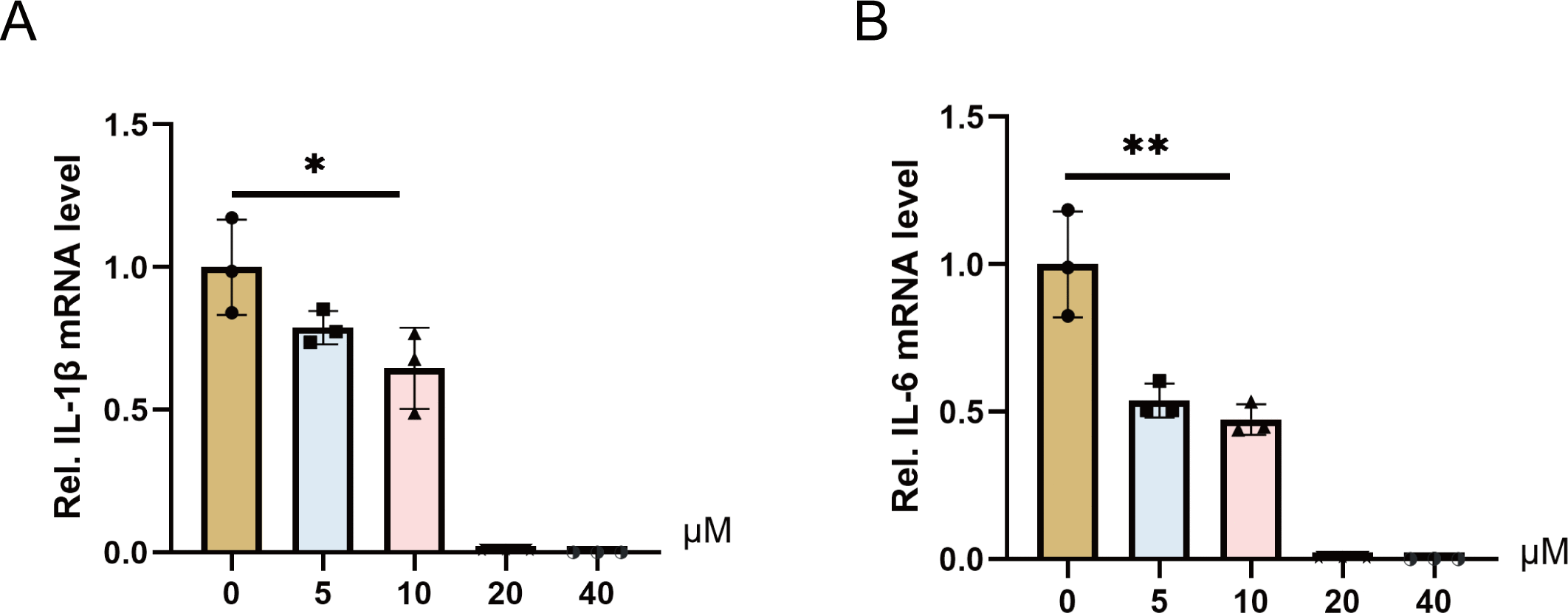

Supplement: S3 Fig — Related to Fig 2C. (A, B) WT peritoneal macrophages were treated with increasing concentrations of the histone H2A ubiquitination inhibitor PRT4165 for 4 h, followed by LPS stimulation for 4 h. IL-1β and IL-6 mRNA expression was quantified by RT-PCR. The data are expressed as means ± SDs. Statistical analysis was carried out using one-way ANOVA. The data were considered statistically significant when P ≤ 0.05 (*), P ≤ 0.01 (**). https://doi.org/10.6084/m9.figshare.30581573. (TIF) [file ppat.1013935.s003.tif]

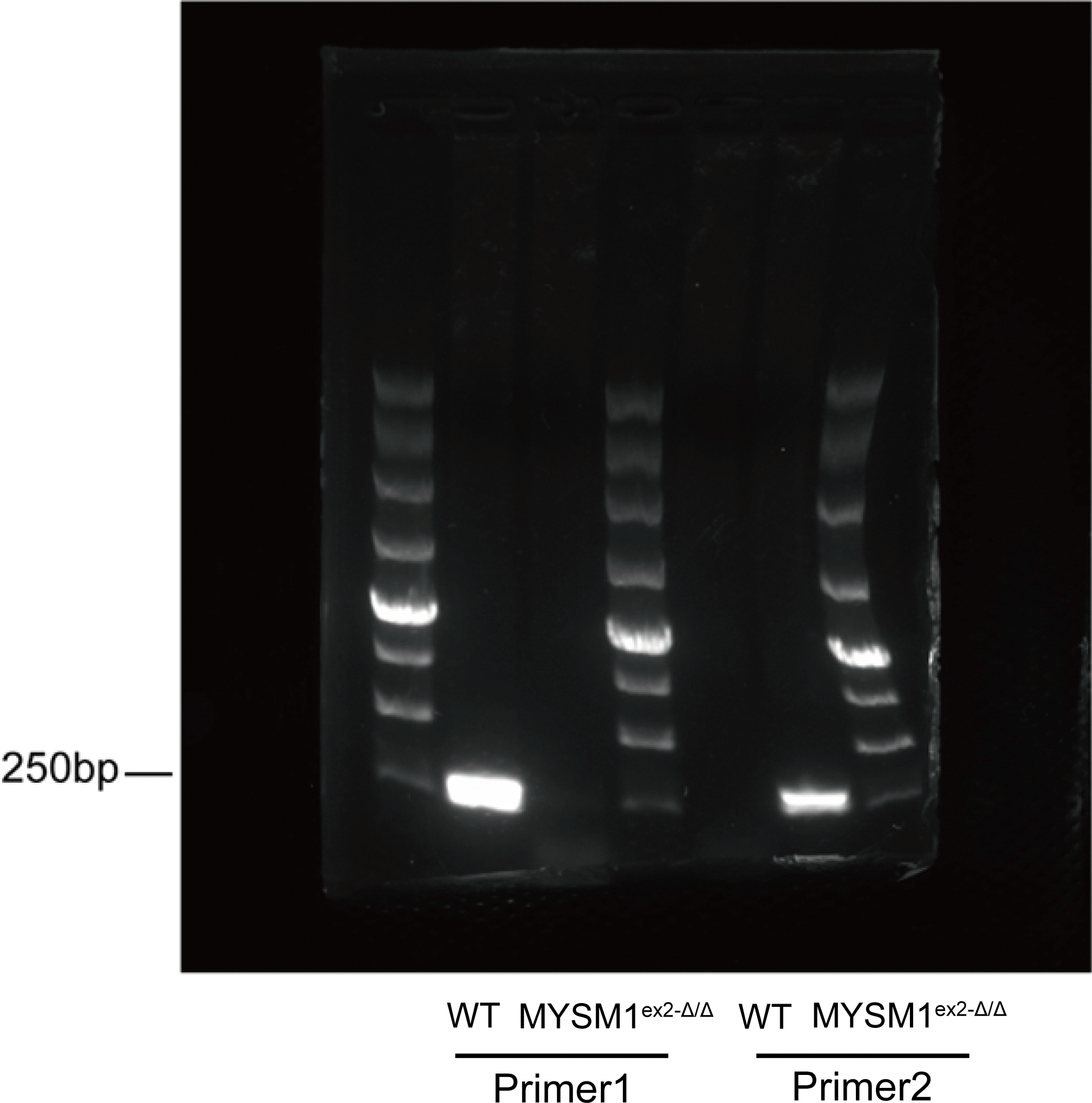

Supplement: S4 Fig — Genotyping was performed on tail tissues excised from WT and MYSM1ex2-Δ/Δ mice. The qPCR primers were as S1 Table. https://doi.org/10.6084/m9.figshare.30581582. (TIF) [file ppat.1013935.s004.tif]

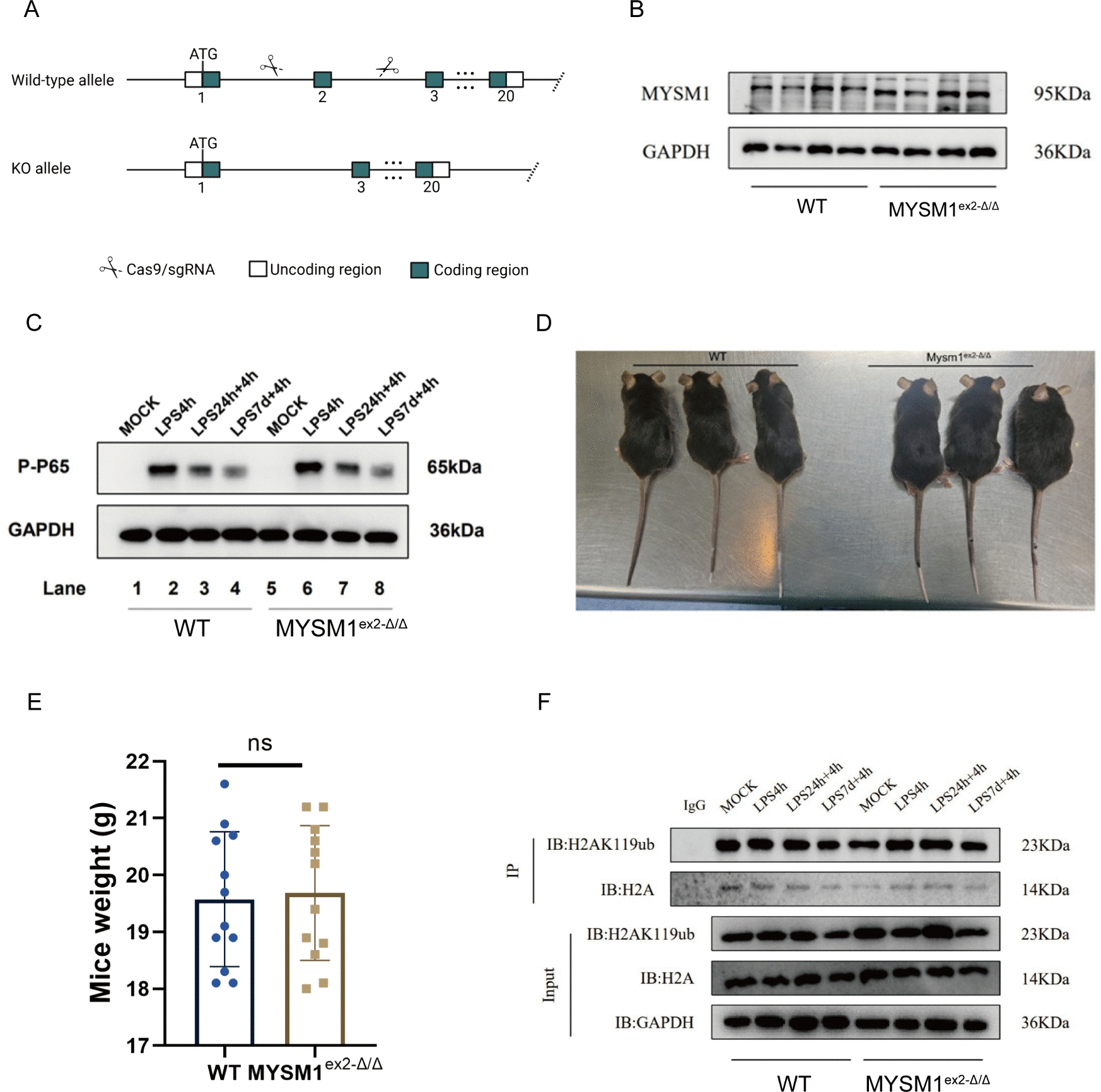

Supplement: S5 Fig — (A) Schematic diagram of the CRISPR/Cas9-based design strategy for generating MYSM1ex2-Δ/Δ mice. S5A Fig: Citation to Use: Created in BioRender. An, Q. (2026) https://BioRender.com/7eganov. (B) MYSM1 protein levels in peritoneal macrophages from WT and MYSM1ex2-Δ/Δ mice were detected by WB. (C) In the LPS-induced secondary infection cell model of sepsis using PMs derived from WT and MYSM1ex2-Δ/Δ mice, P-P65 protein levels were detected by WB. (D) Representative gross appearance of WT and MYSM1ex2-Δ/Δ mice. (E) Body weight of WT(n = 12) and MYSM1ex2-Δ/Δ mice(n = 12). (F) In the LPS-induced secondary infection cell model of sepsis using PMs derived from WT and MYSM1ex2-Δ/Δ mice, immunoprecipitation (Co-IP) and western blot (WB) analysis were performed to validate the specificity of the H2AK119ub antibody. https://doi.org/10.6084/m9.figshare.30581600. (TIF) [file ppat.1013935.s005.tif]

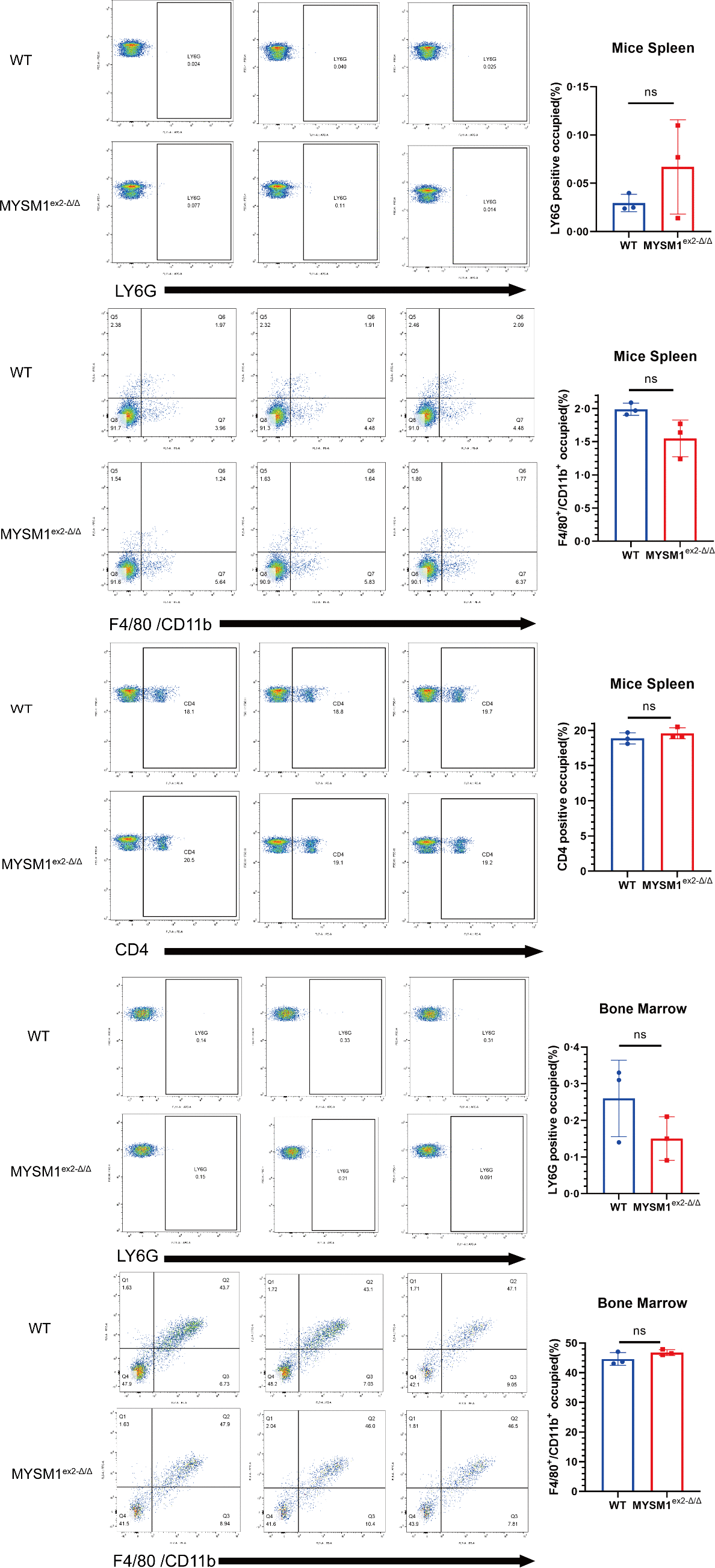

Supplement: S6 Fig — Flow cytometry analysis of immune cell composition in the spleens or bone marrow from WT mice (n = 3) and MYSM1ex2-Δ/Δ mice (n = 3). The data were expressed as means ± SDs. The statistical analysis was carried out using the t test. The data were considered not statistically significant when (p ＞ 0.05). https://doi.org/10.6084/m9.figshare.30581633. (TIF) [file ppat.1013935.s006.tif]

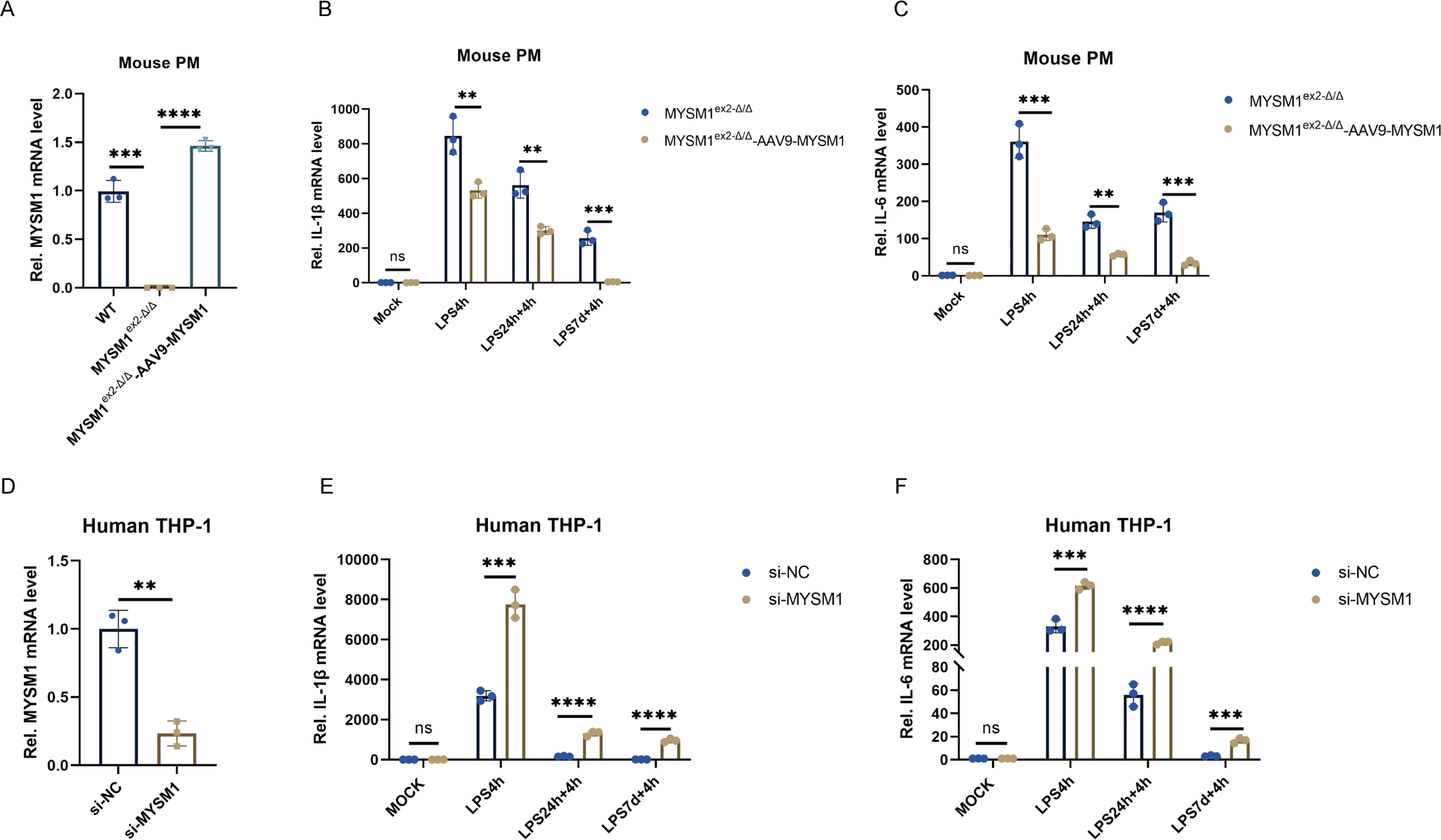

Supplement: S7 Fig — (A) Peritoneal macrophages were isolated from WT and MYSM1ex2-Δ/Δ mice, transduced with AAV9 to overexpress MYSM1, and MYSM1 mRNA expression was assessed by RT-PCR. (B, C) Peritoneal macrophages isolated from MYSM1ex2-Δ/Δ mice were transduced with AAV9 to overexpress MYSM1, followed by LPS stimulation to model secondary infection. The transcriptional levels of IL-1β and IL-6 were then assessed by RT-PCR. (D) MYSM1 was knocked down in THP-1 cells using siRNA, and MYSM1 mRNA expression was assessed by RT-PCR. (E, F) MYSM1 was knocked down in THP-1 cells using siRNA, followed by LPS stimulation to model secondary infection, and IL-1β and IL-6 mRNA levels were measured by RT-PCR. https://doi.org/10.6084/m9.figshare.31101058. (TIF) [file ppat.1013935.s007.tif]
