# Supplementary material for: MYSM1-mediated epigenetic modification dysregulation leads to immunosuppression and secondary infections in sepsis
Source: PLoS Pathog. 2026 Feb 12;22(2):e1013935. doi: 10.1371/journal.ppat.1013935 (PMC12928581; doi:10.1371/journal.ppat.1013935)
Supplement: S3 Data — https://doi.org/10.6084/m9.figshare.30581675. (DOCX) [file ppat.1013935.s014.docx]

**MYSM1-mediated epigenetic modification dysregulation leads to immunosuppression and secondary infections in sepsis**

Jiali Xiong **^1*^**，Xin Cheng **^1*^**，Xiaoxing Xiong **^2^**, Heyang Zhang **^5^**，Qi An**^6^**, Zhiqiang Li **^7^**, Hong Fan**^7^** , Guangli Li **^8^**, Wei Li **^3#^**, Mingfu Tian **^4#^**, Jingjun Lv **^1#^**

**^1^** Department of Emergency, Renmin Hospital of Wuhan University, Wuhan, Hubei 430060, PR China

**^2^** Department of Neurosurgery, Renmin Hospital of Wuhan University, Wuhan, Hubei 430060, PR China

**^3^**Department of Anesthesiology, Renmin Hospital of Wuhan University, Wuhan, Hubei 430060, PR China

**^4^**State Key Laboratory of Virology, College of Life Sciences, Wuhan University, Wuhan, Hubei 430072, PR China

**^5^** Department of Gastroenterology, Beijing Friendship Hospital, Capital Medical University, 100050, Beijing, China.

**^6^**Department of Oncology, Renmin Hospital of Wuhan University, Wuhan, 430060, China.

**^7^**Department of Clinical Laboratory, Institute of Translational Medicine, Renmin Hospital of Wuhan University, Wuhan, Hubei 430060, PR China

**^8^**Postgraduate Training Base at Shanghai Gongli Hospital, Ningxia Medical University, Shanghai, 200135, PR China

*These authors contributed equally to this work.

**^#^**Correspondence: Wei Li [150220995@qq.com](mailto:150220995@qq.com); Mingfu Tian [2015202040056@whu.edu.cn](mailto:2015202040056@whu.edu.cn) ; Jingjun Lv [lvjingjun@whu.edu.cn](mailto:lvjingjun@whu.edu.cn)

**Raw ImageFile from Western Blots**

**Fig2A**


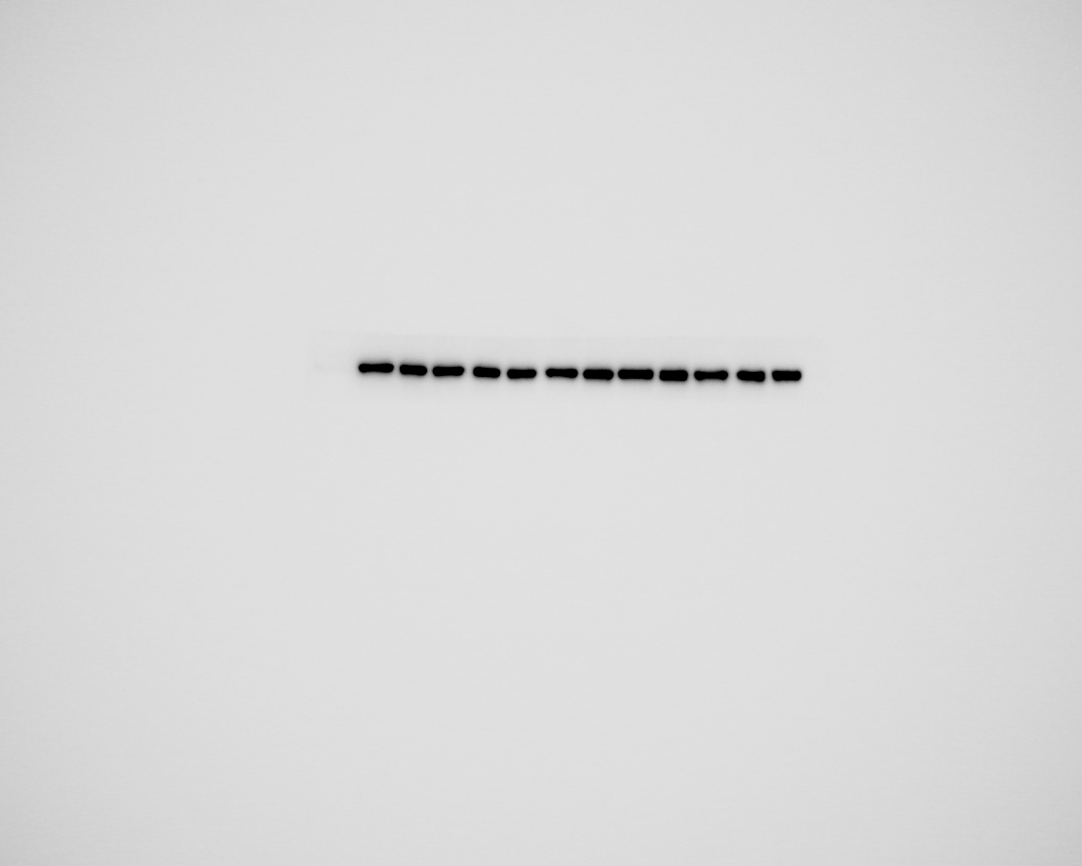


GAPDH


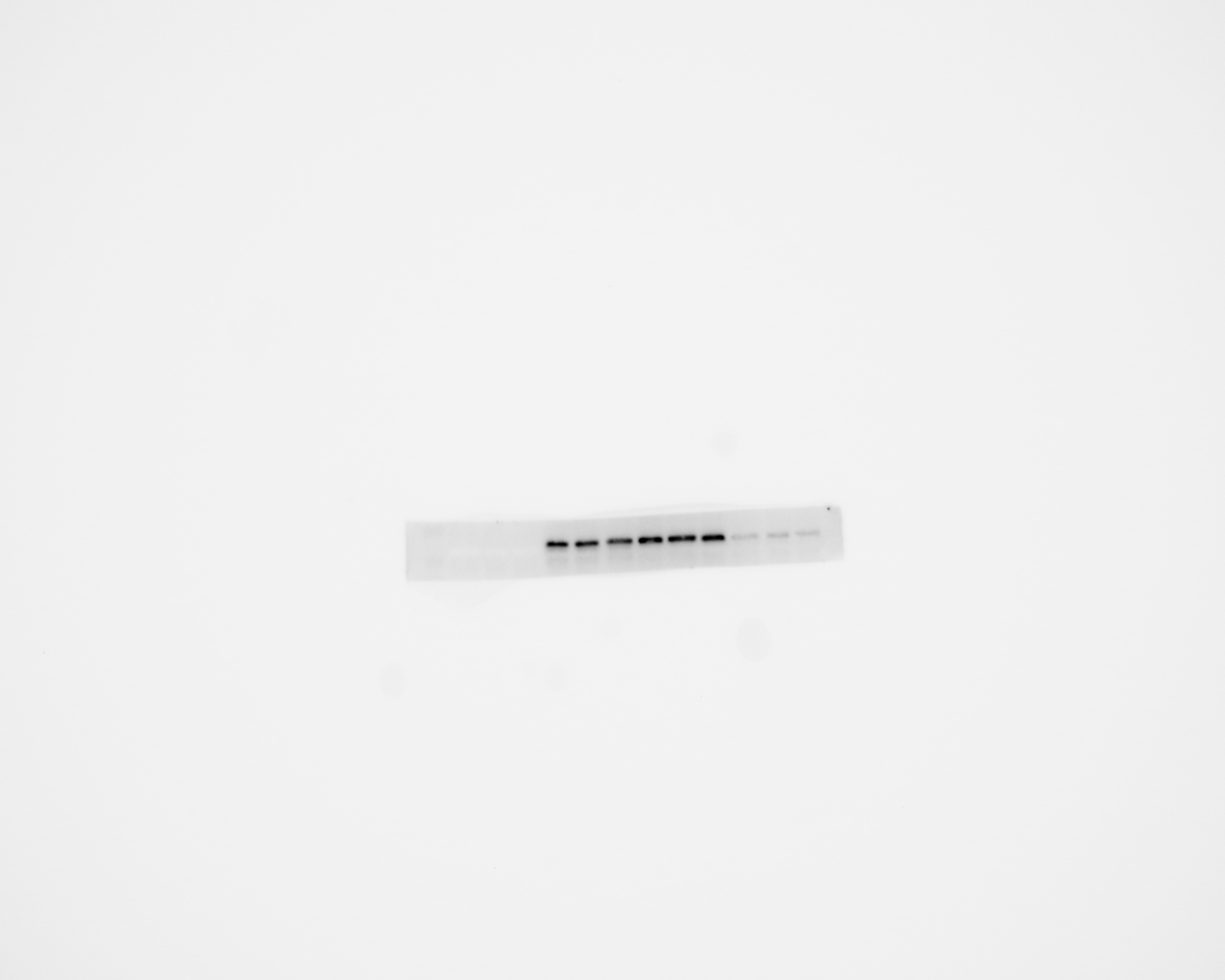


IL-1β


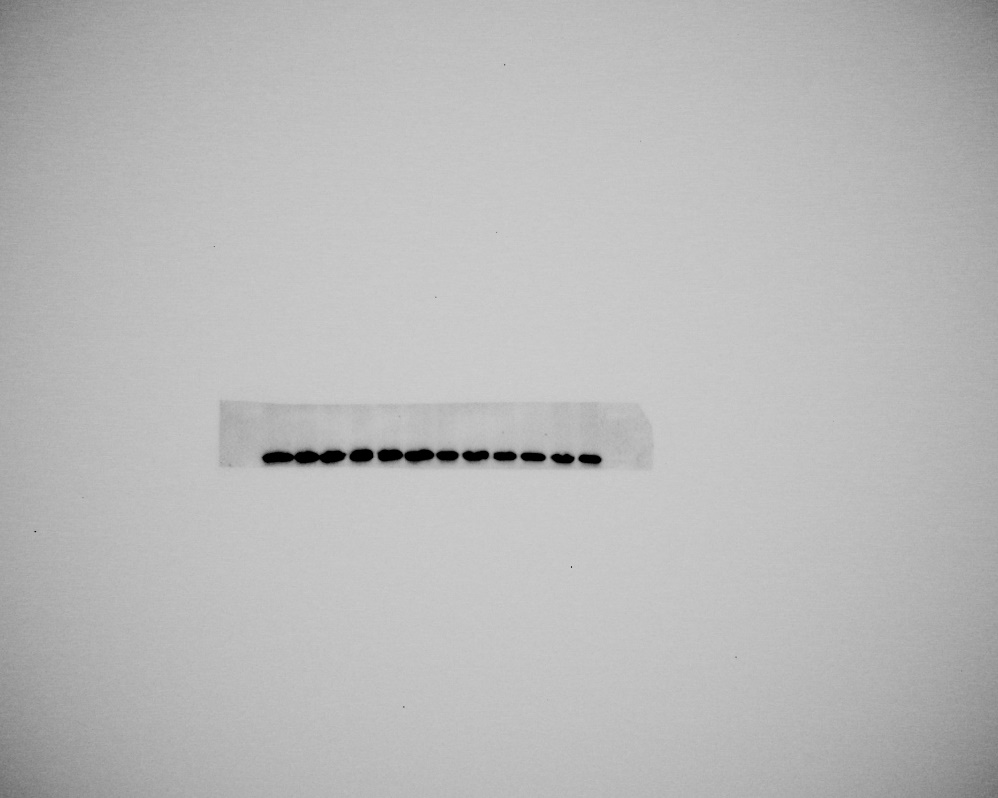


H2AK119ub


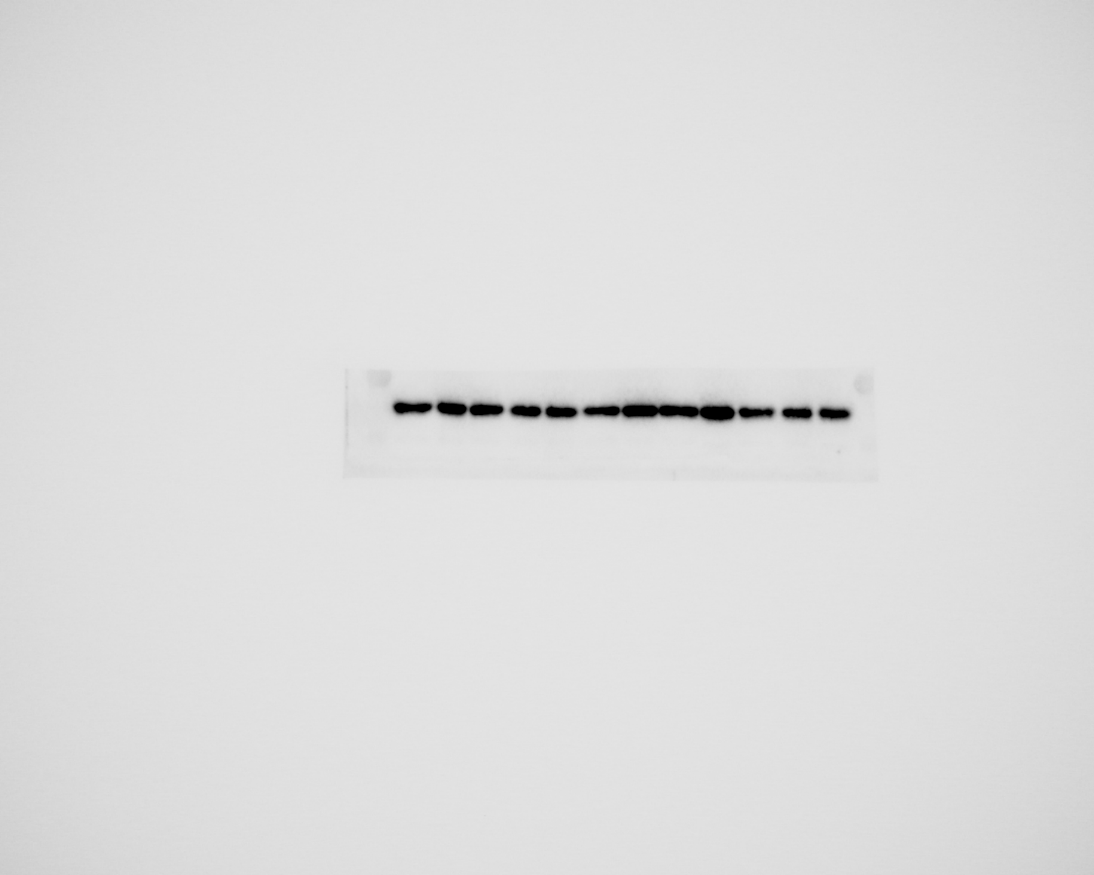


H3K36me3


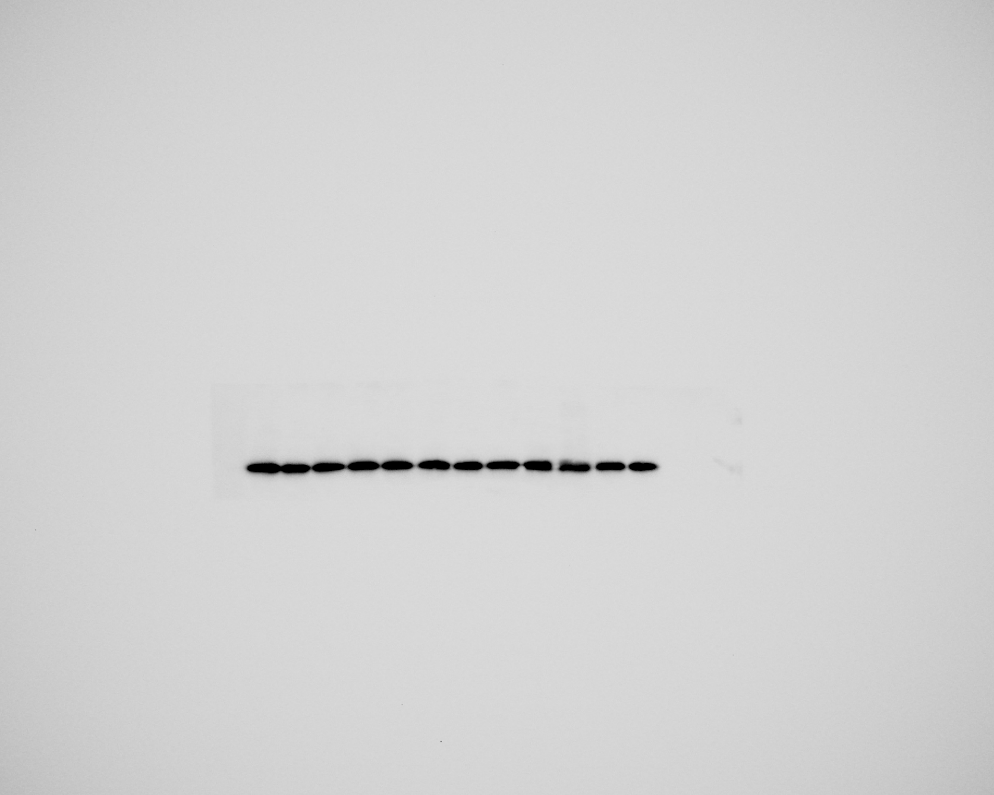


H3K79me3


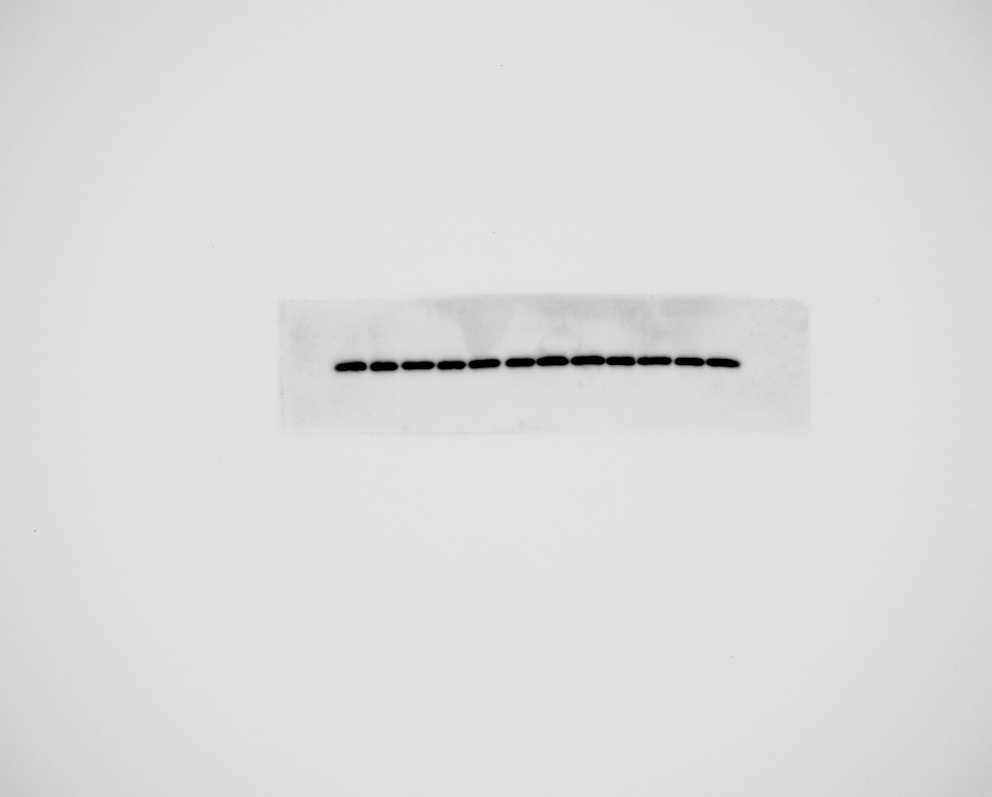


H3K9me3


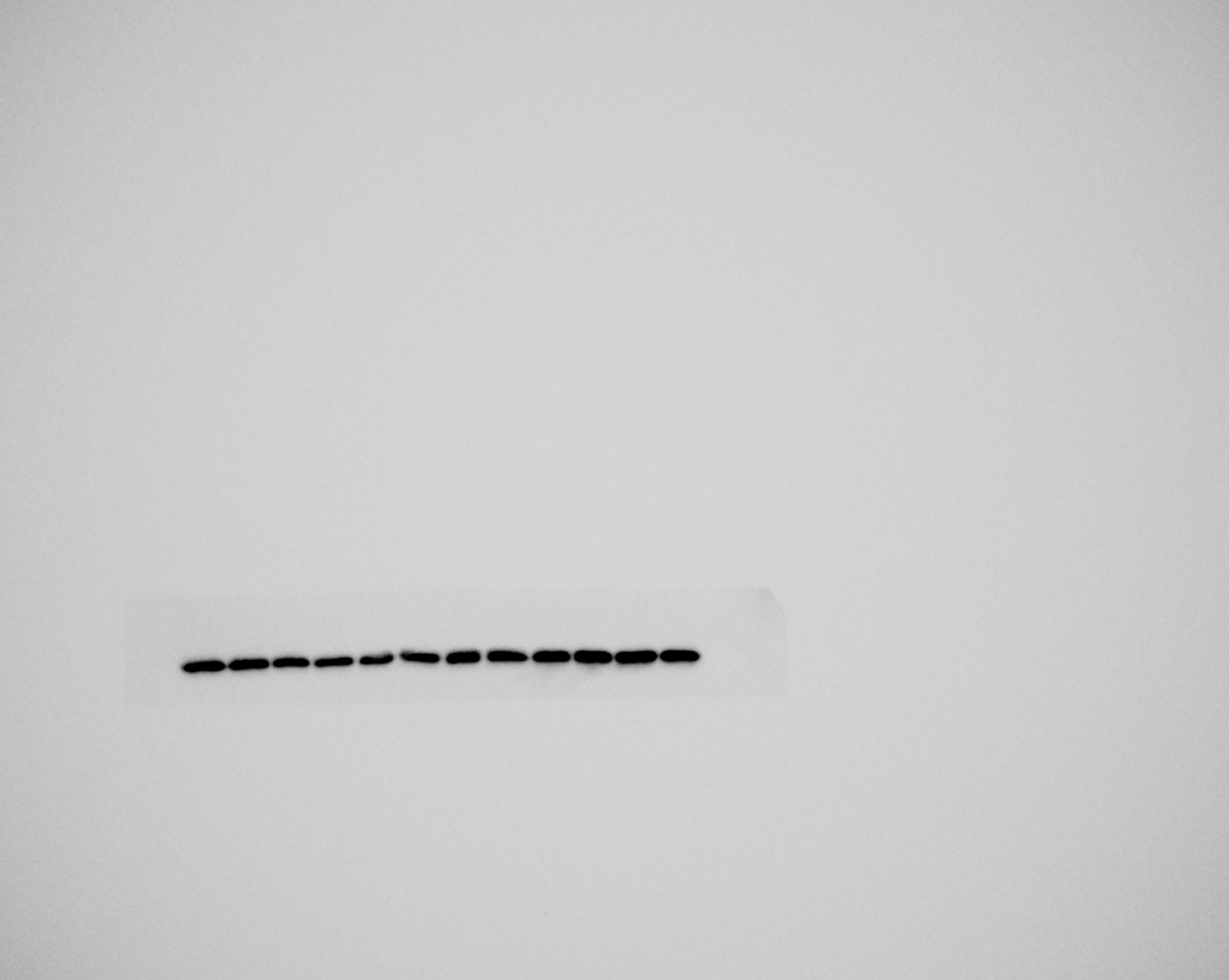


H3K27me3


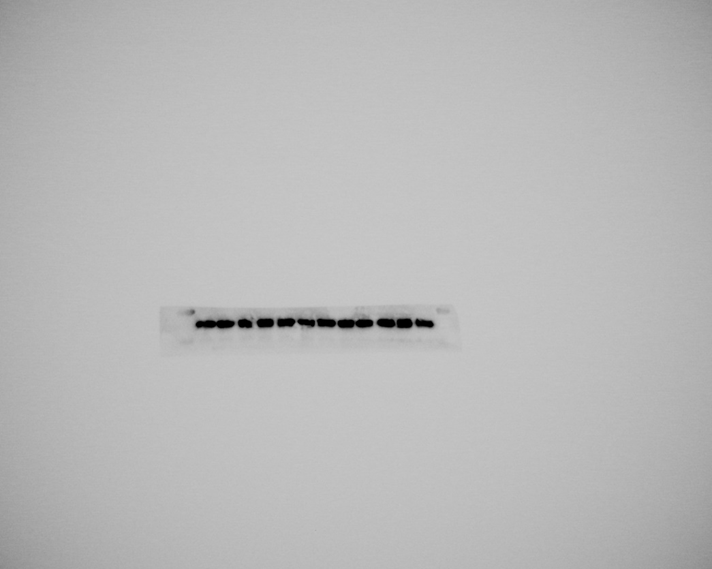


H3K27ac

**Fig2B**


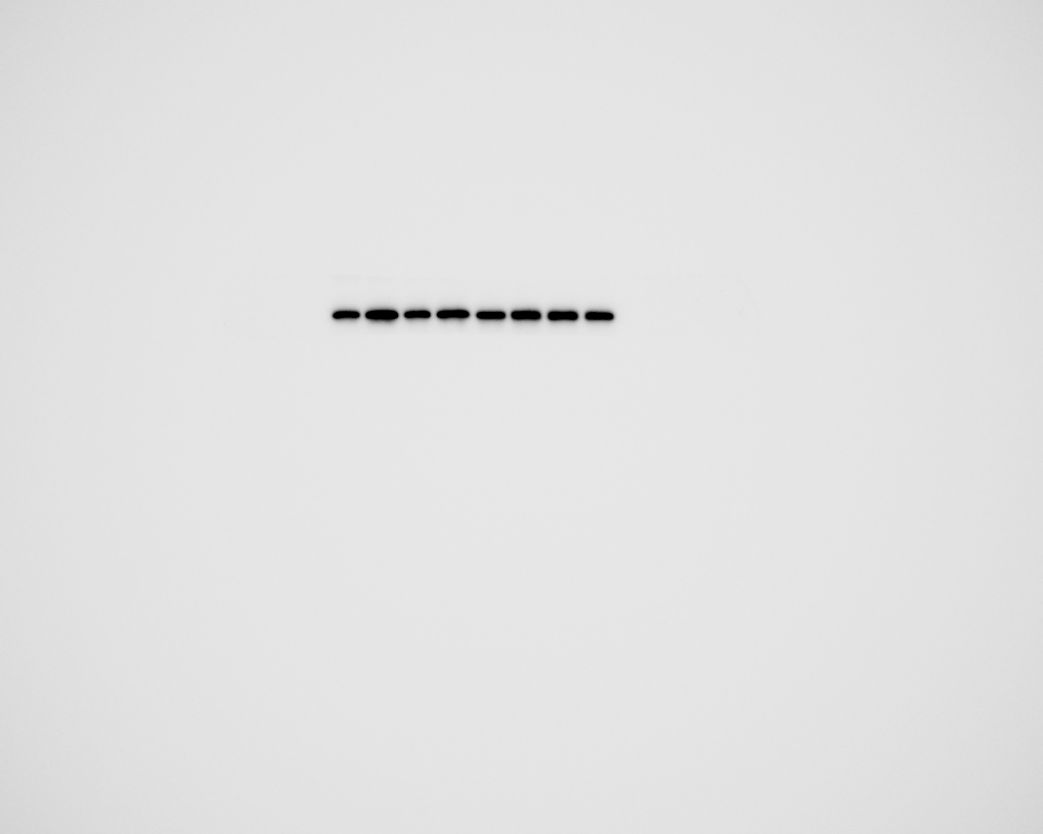


GAPDH


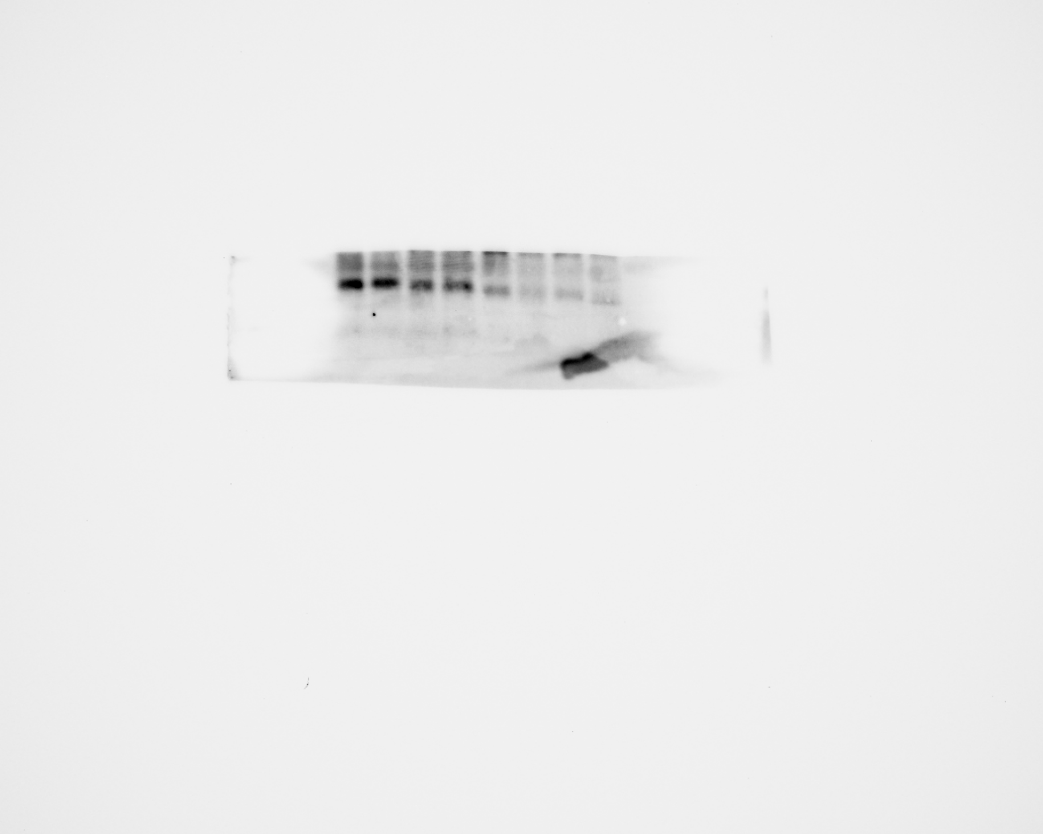


H2AK119ub

**Fig2C**

**
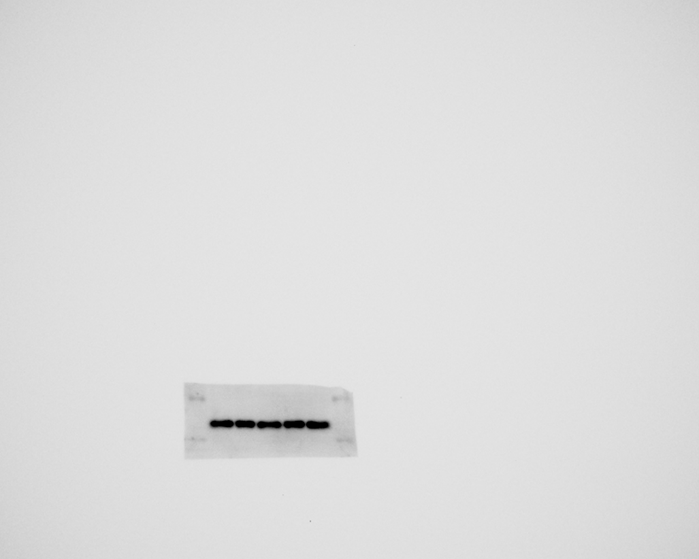
**

GAPDH


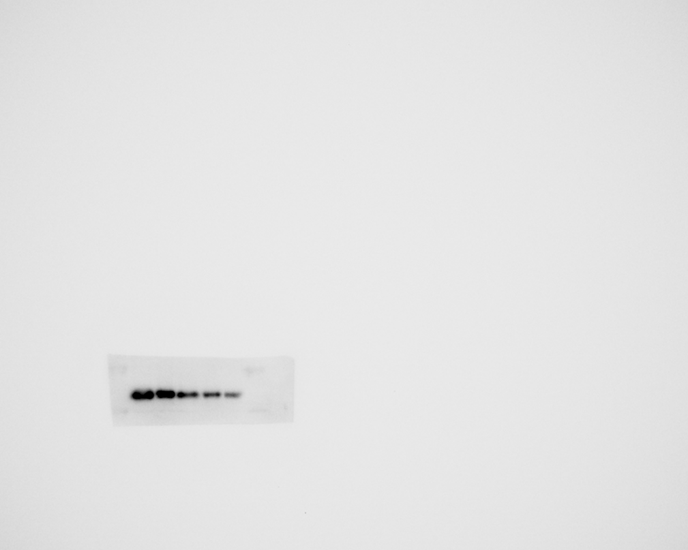


IL-1β


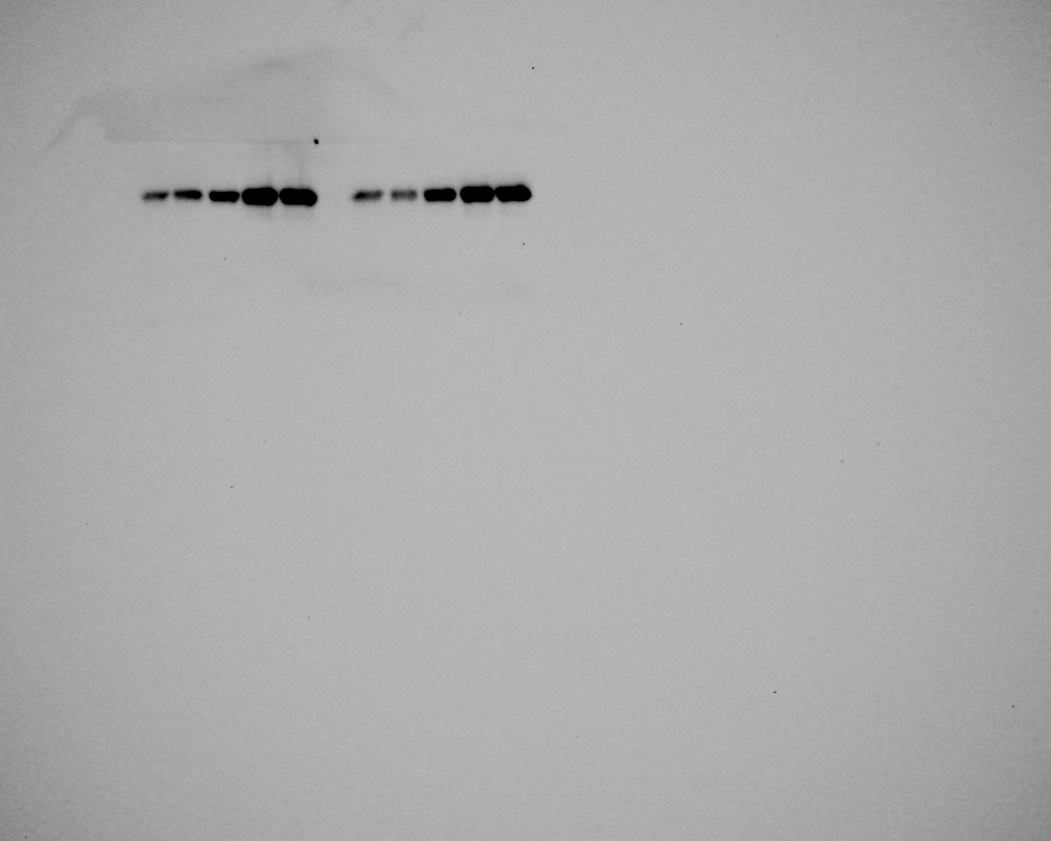


H2AK119ub

**Fig3C**


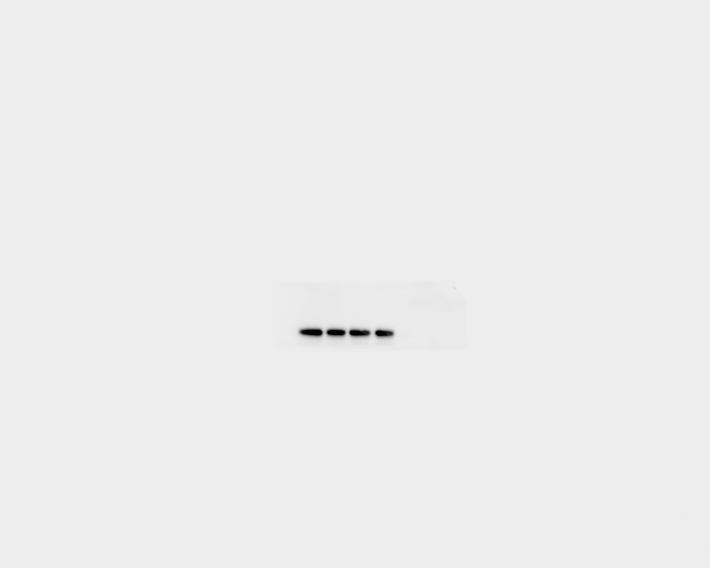


Input GAPDH


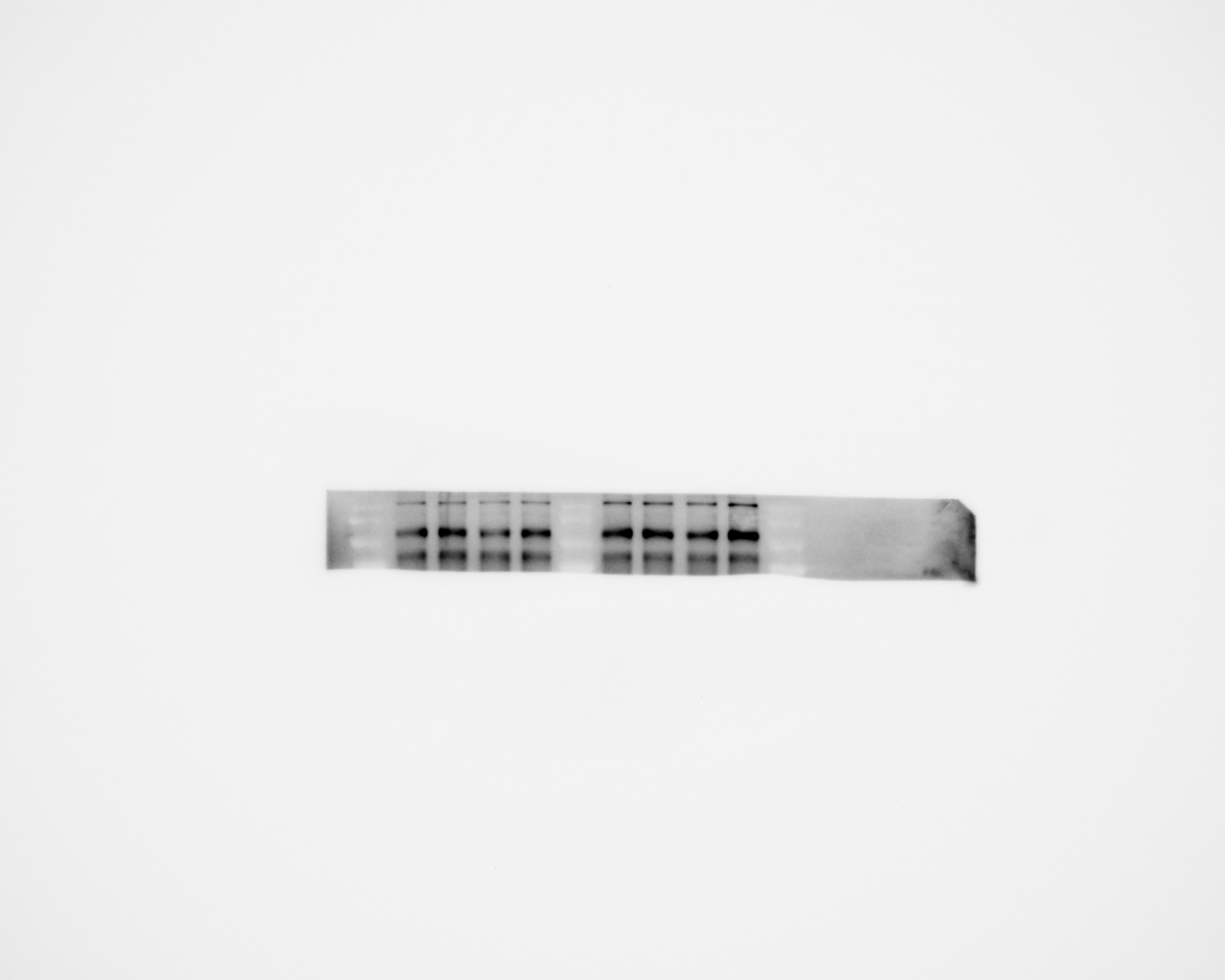


Input MYSM1


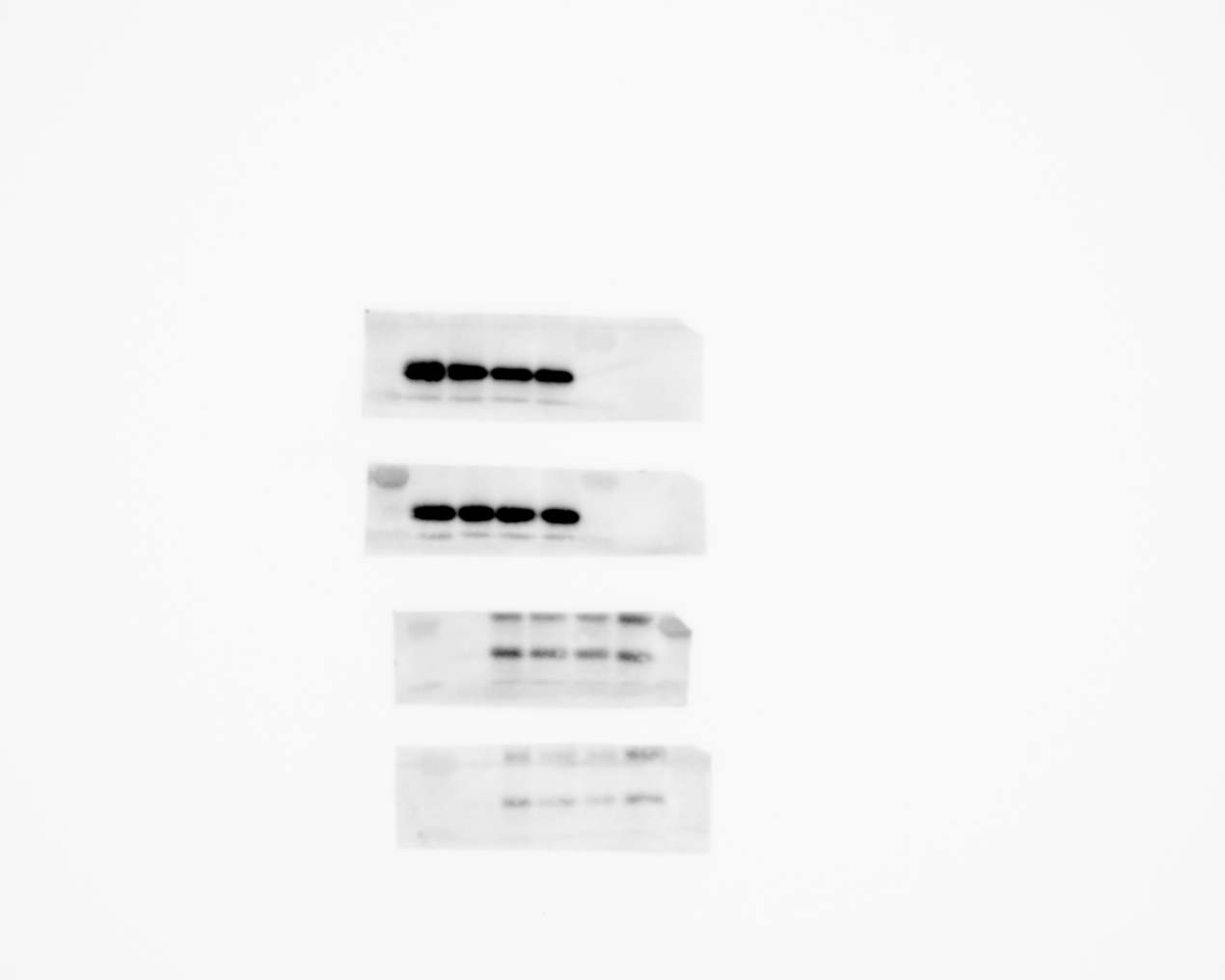


Input H2AK119ub


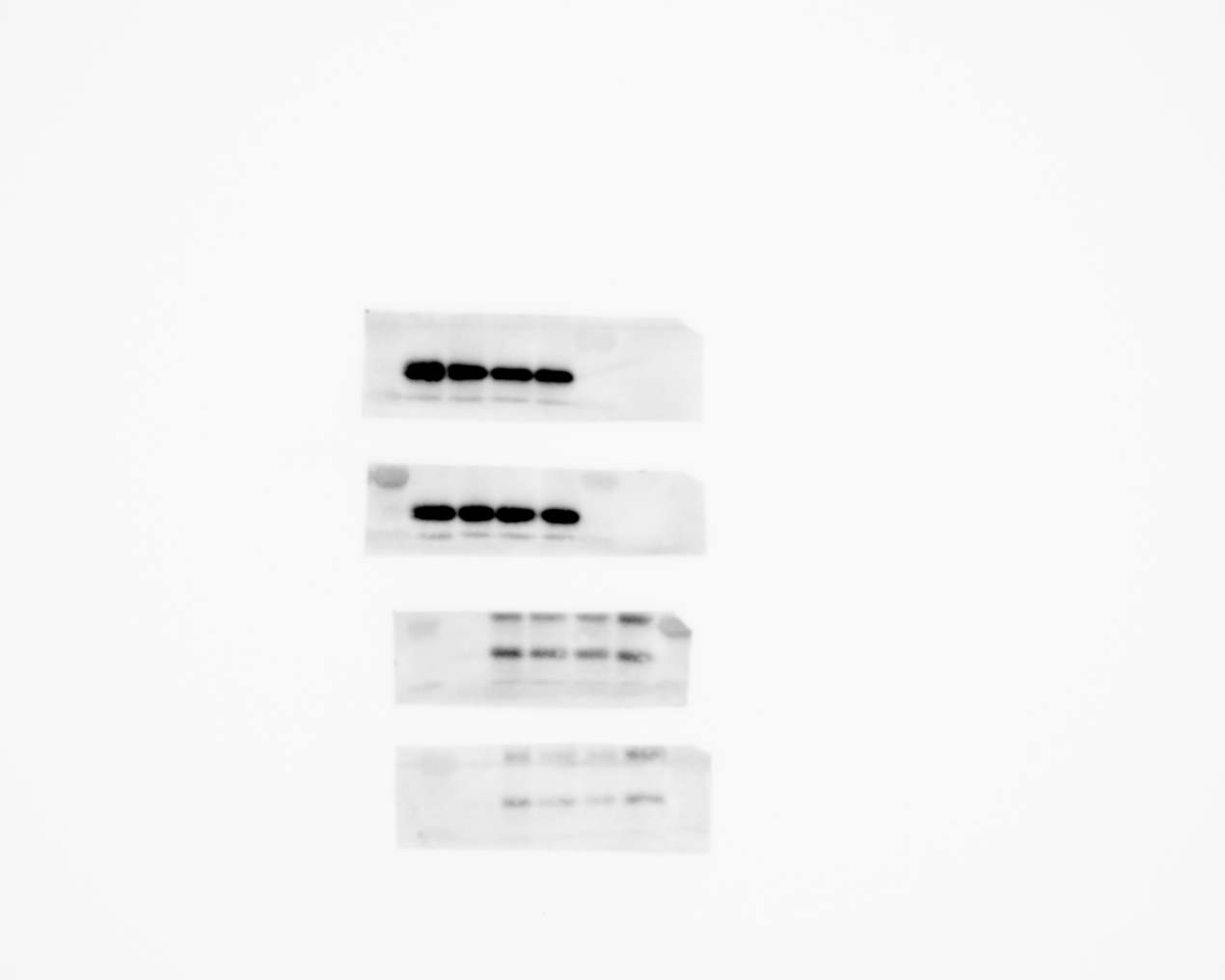


IP H2AK119ub


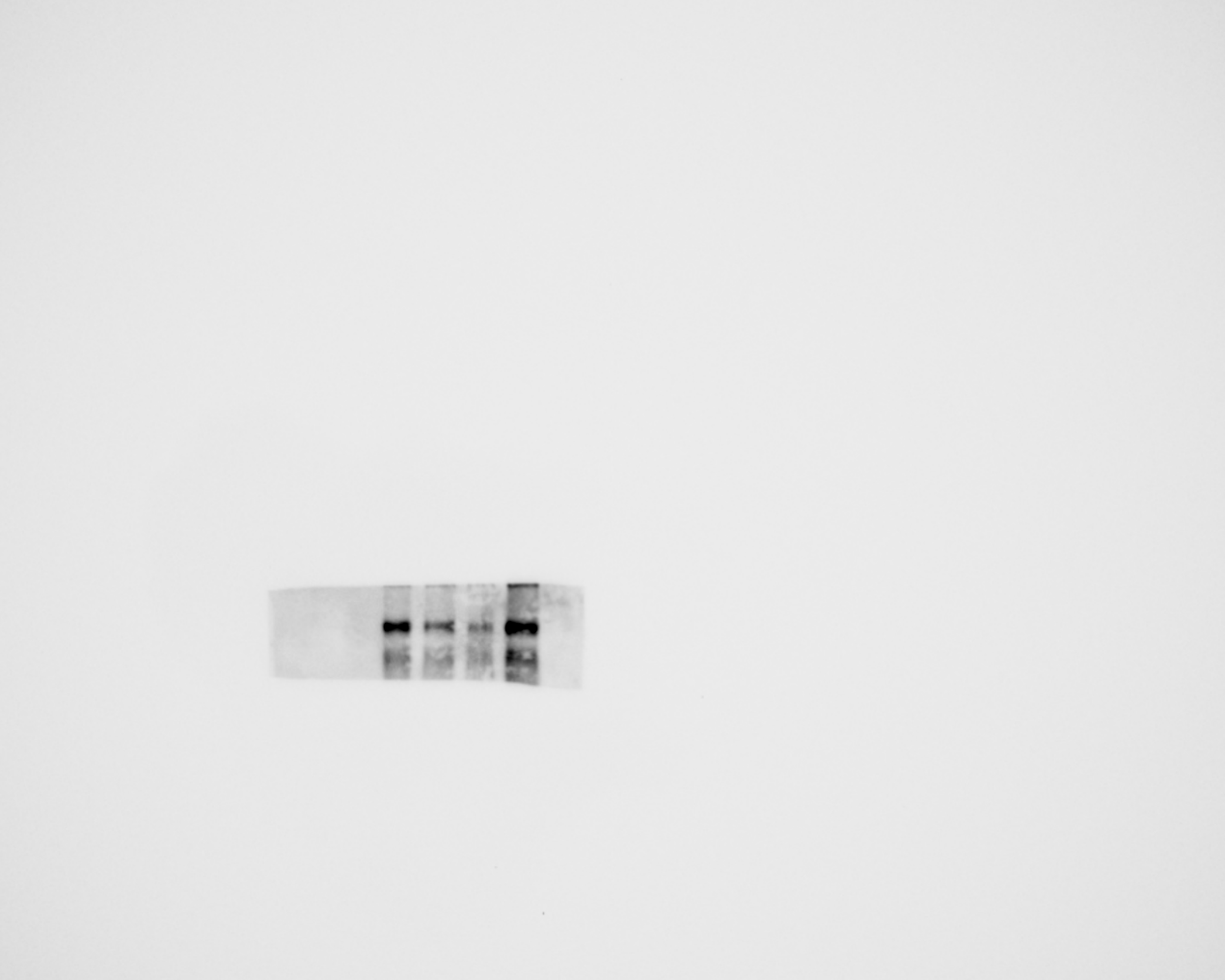


IP MYSM1

**Fig3D**


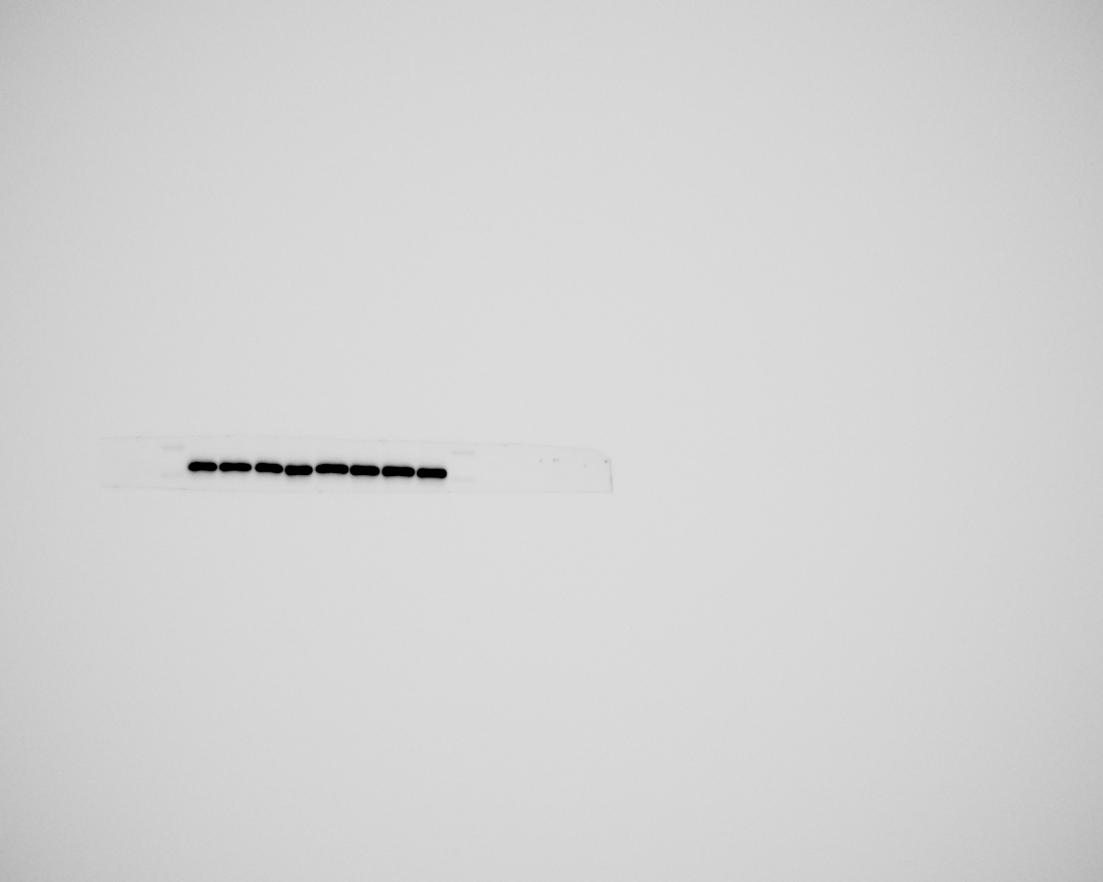


GAPDH


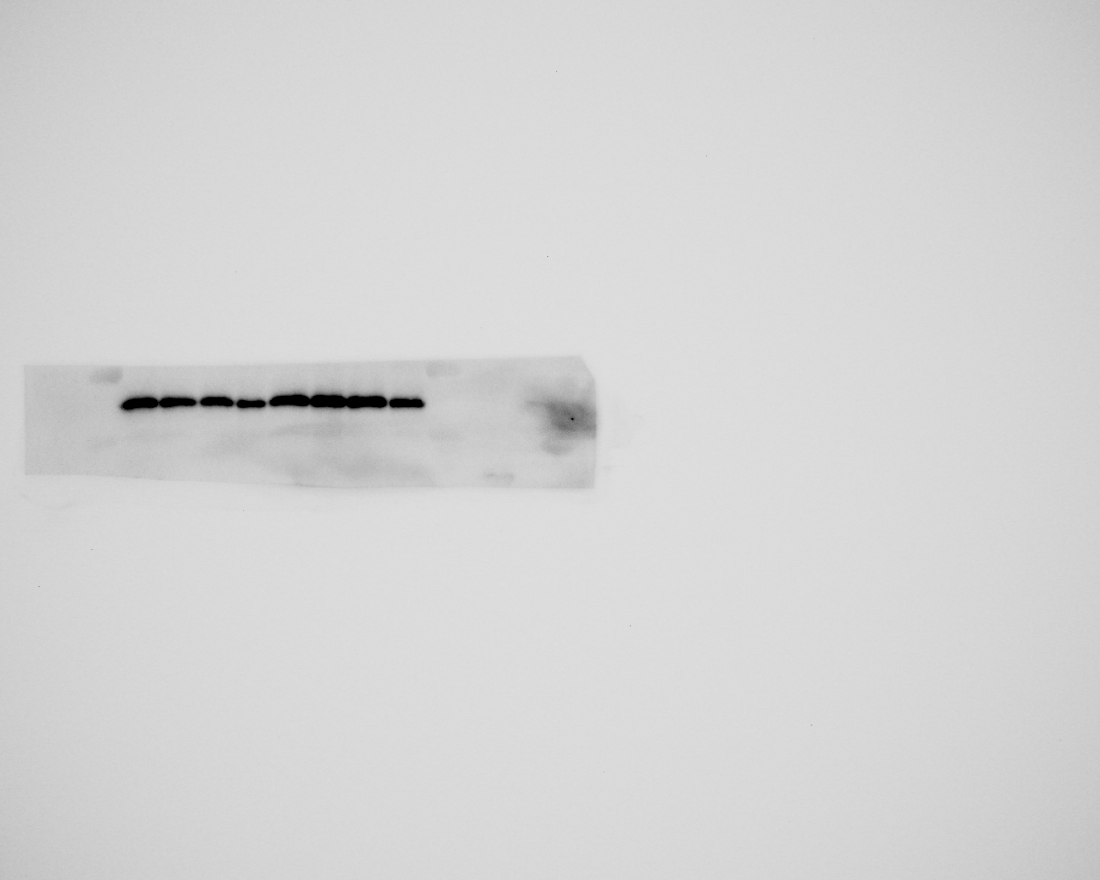


H2AK119ub


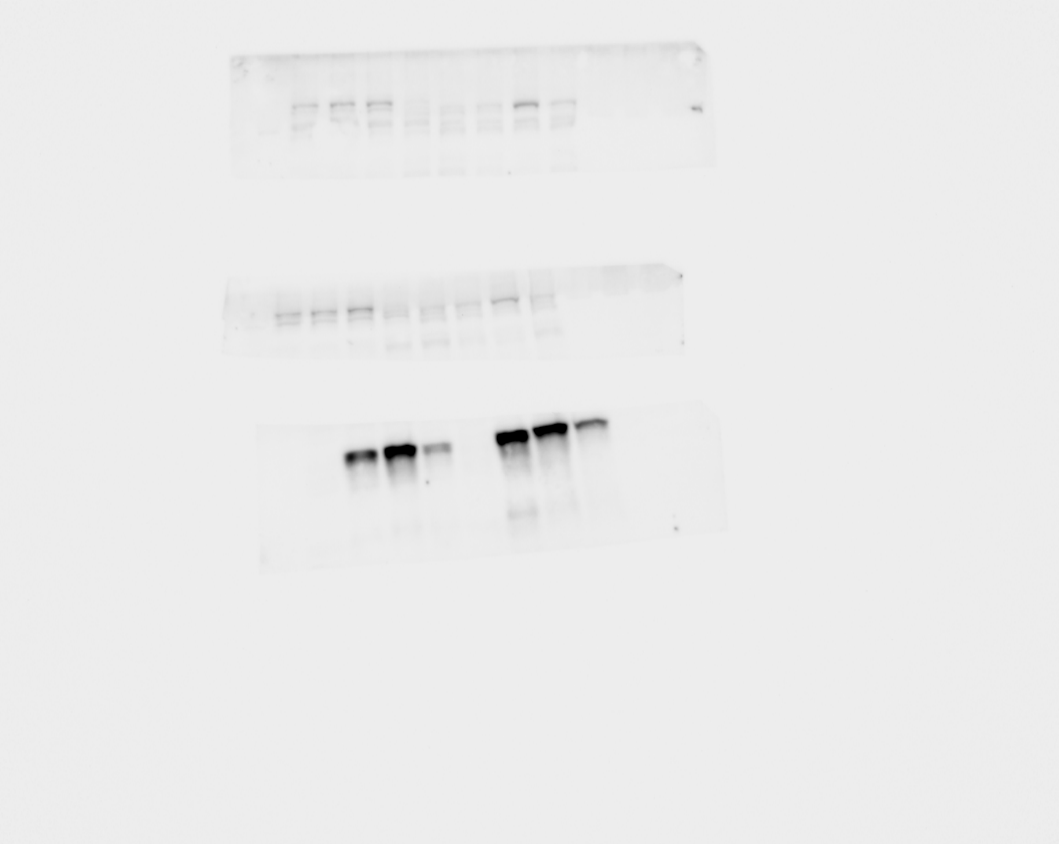


IL-1β

**Fig S5B**


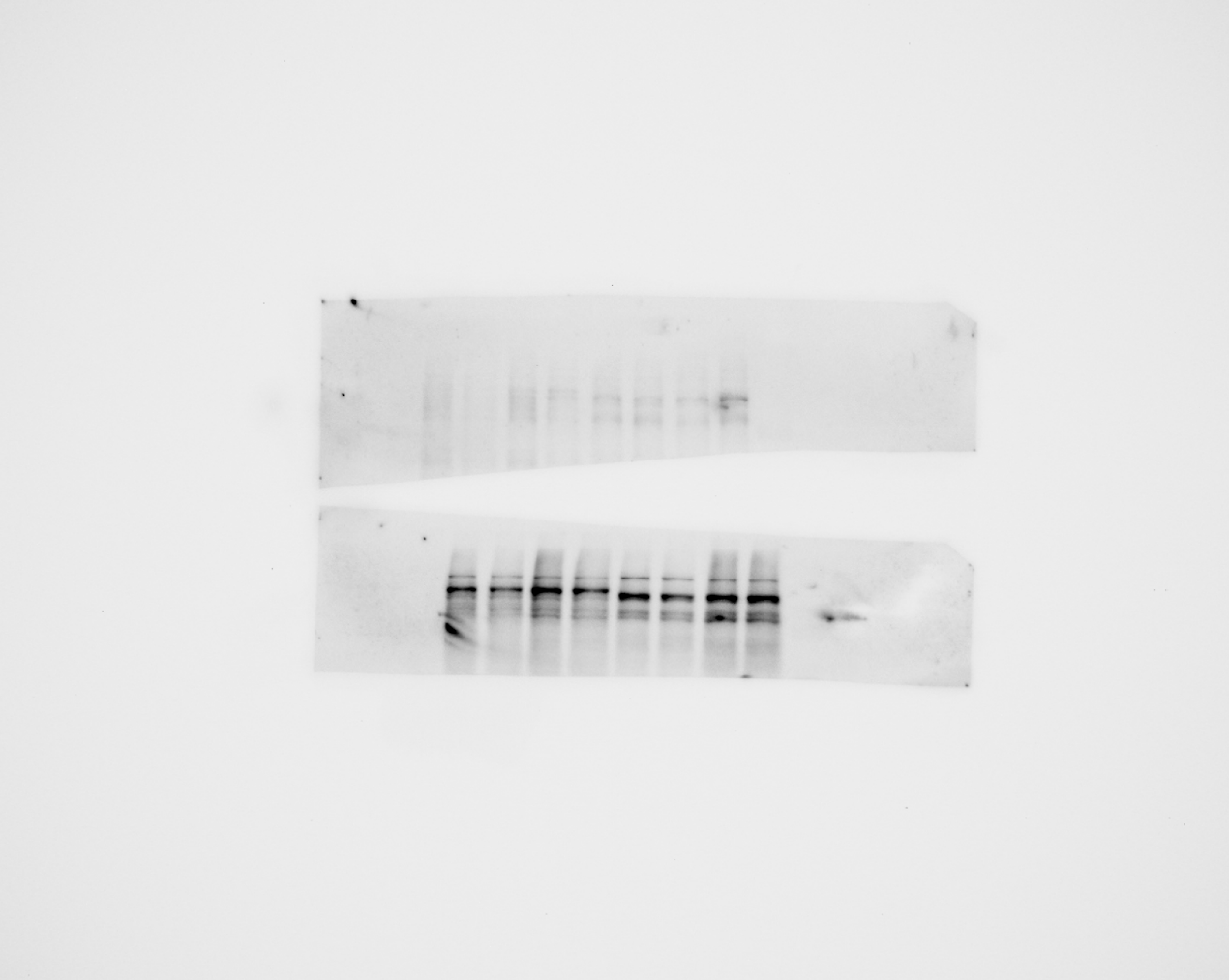


MYSM1


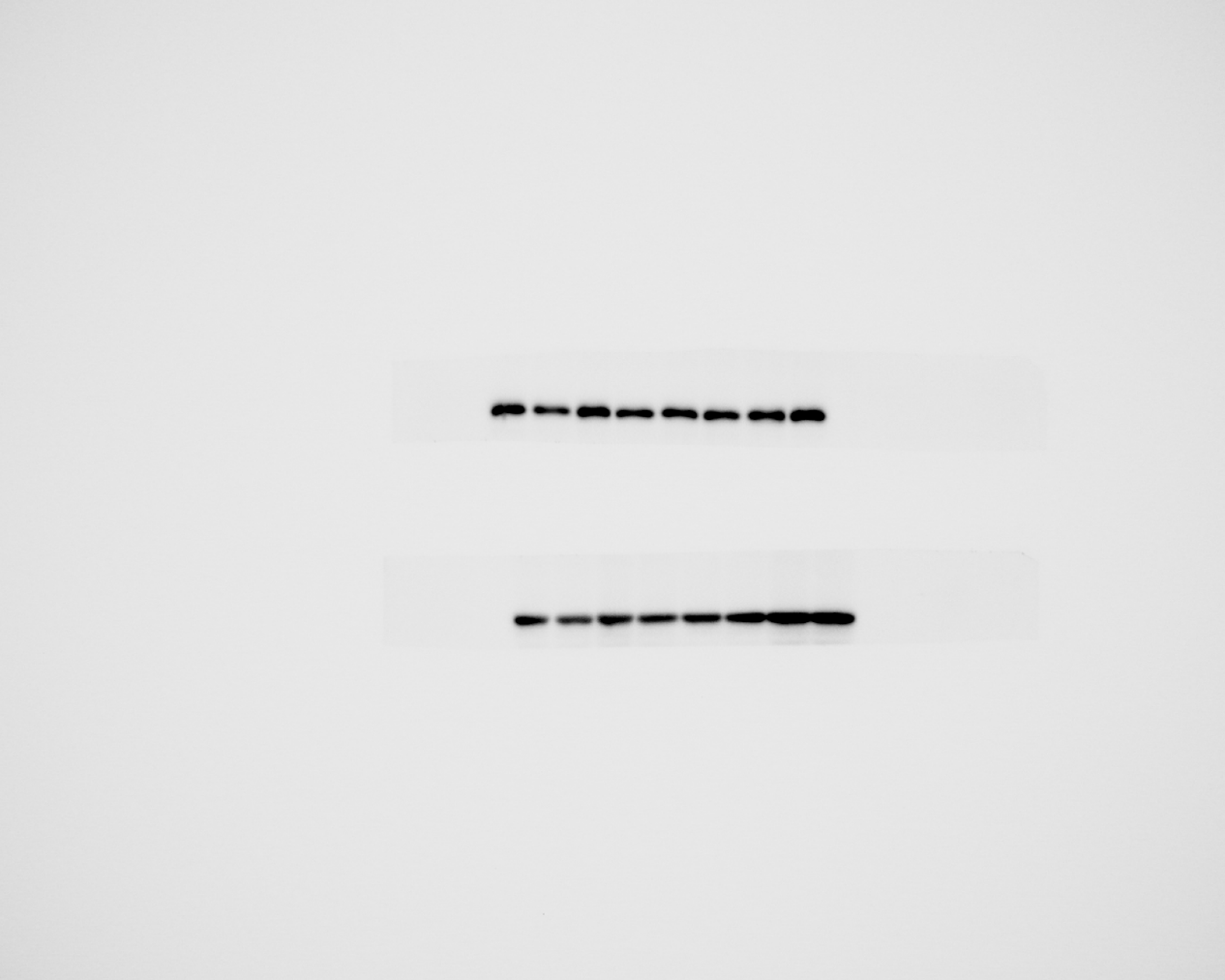


GAPDH

**Fig S5C**


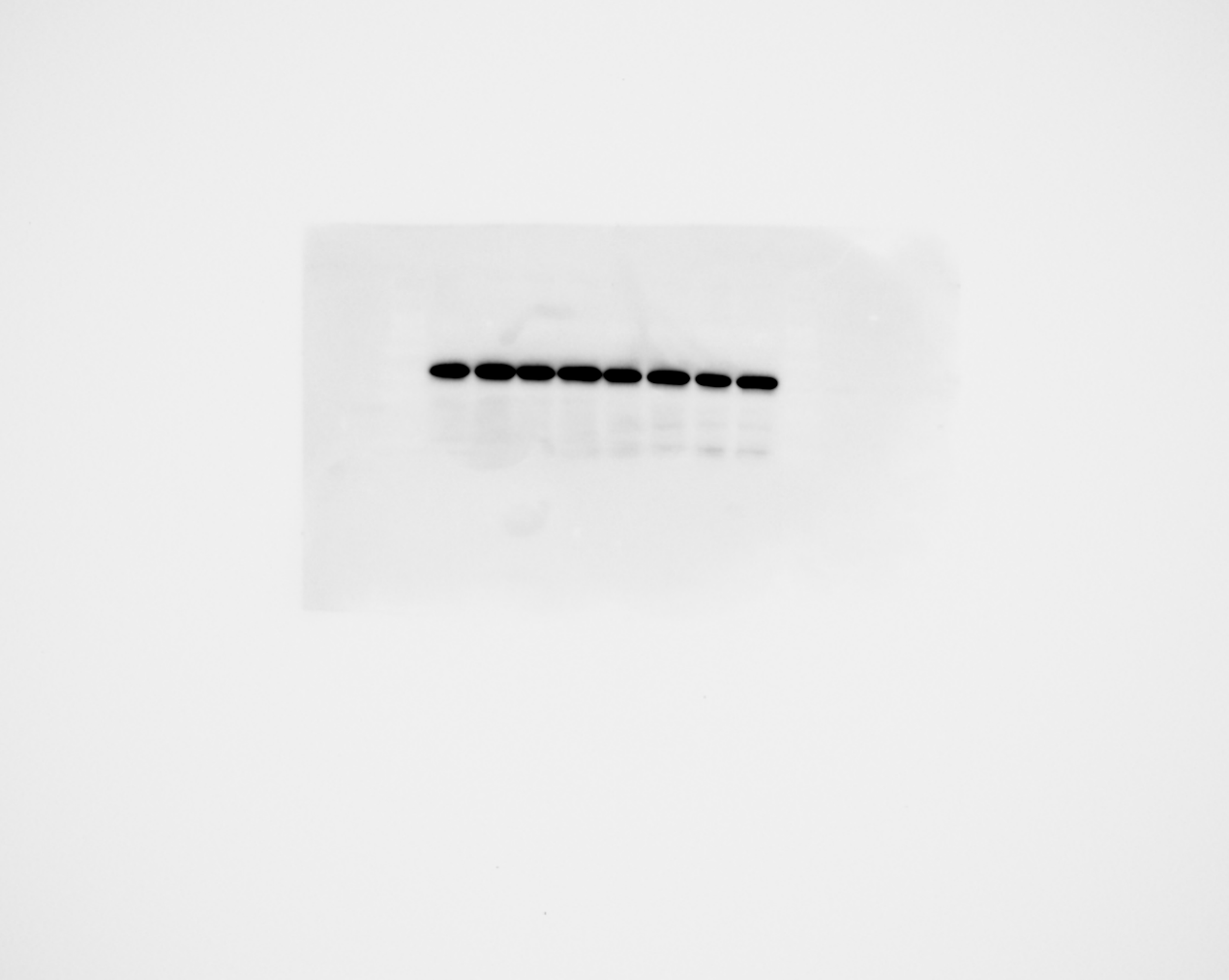


GAPDH

**
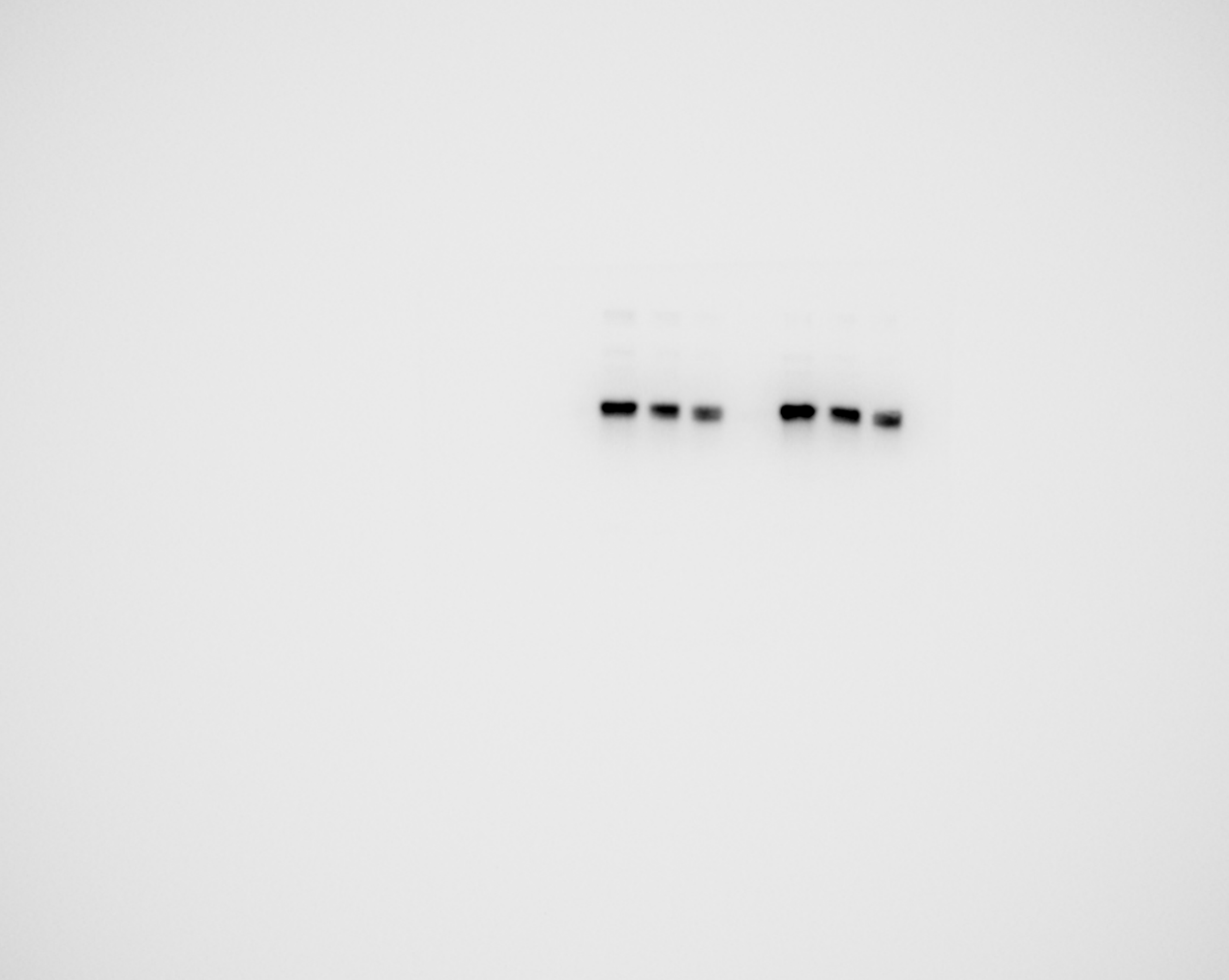
**

P-P65

**Fig S5F**


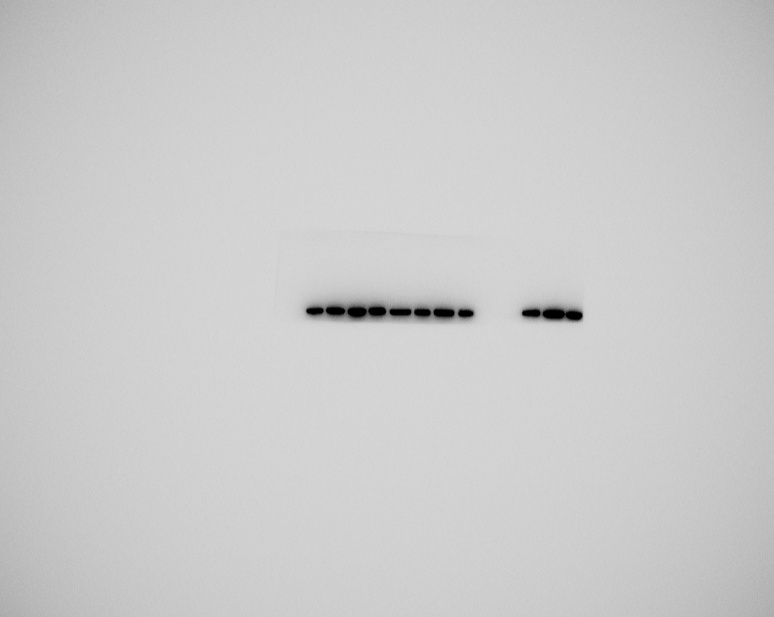


Input GAPDH


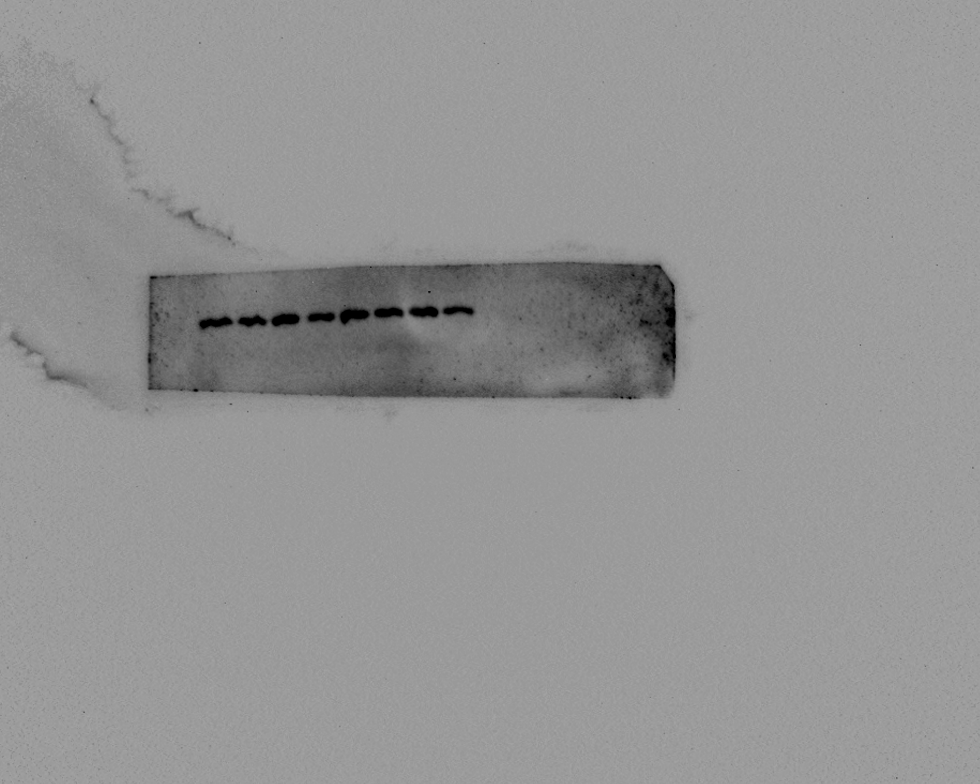


H2A


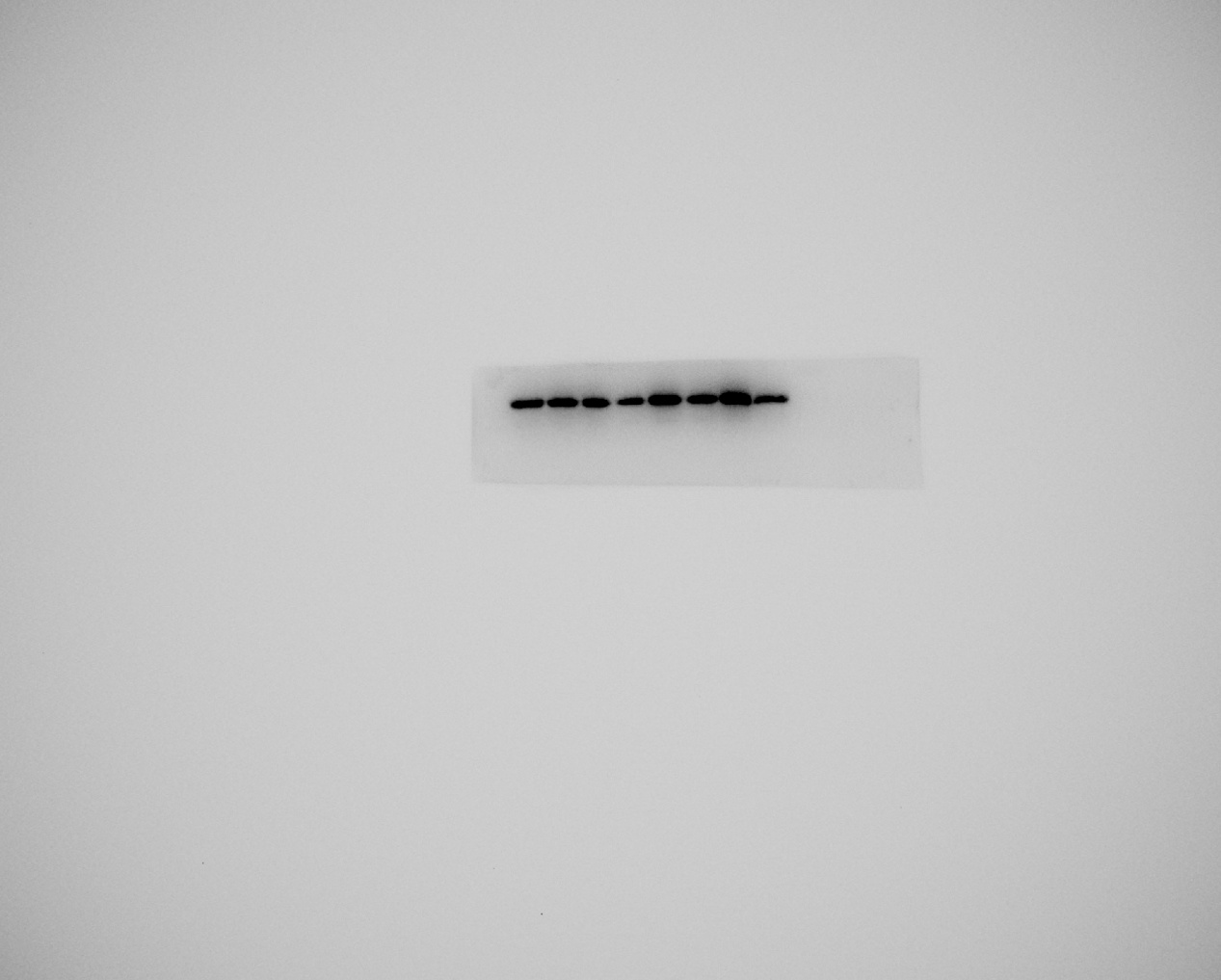


H2AK119ub


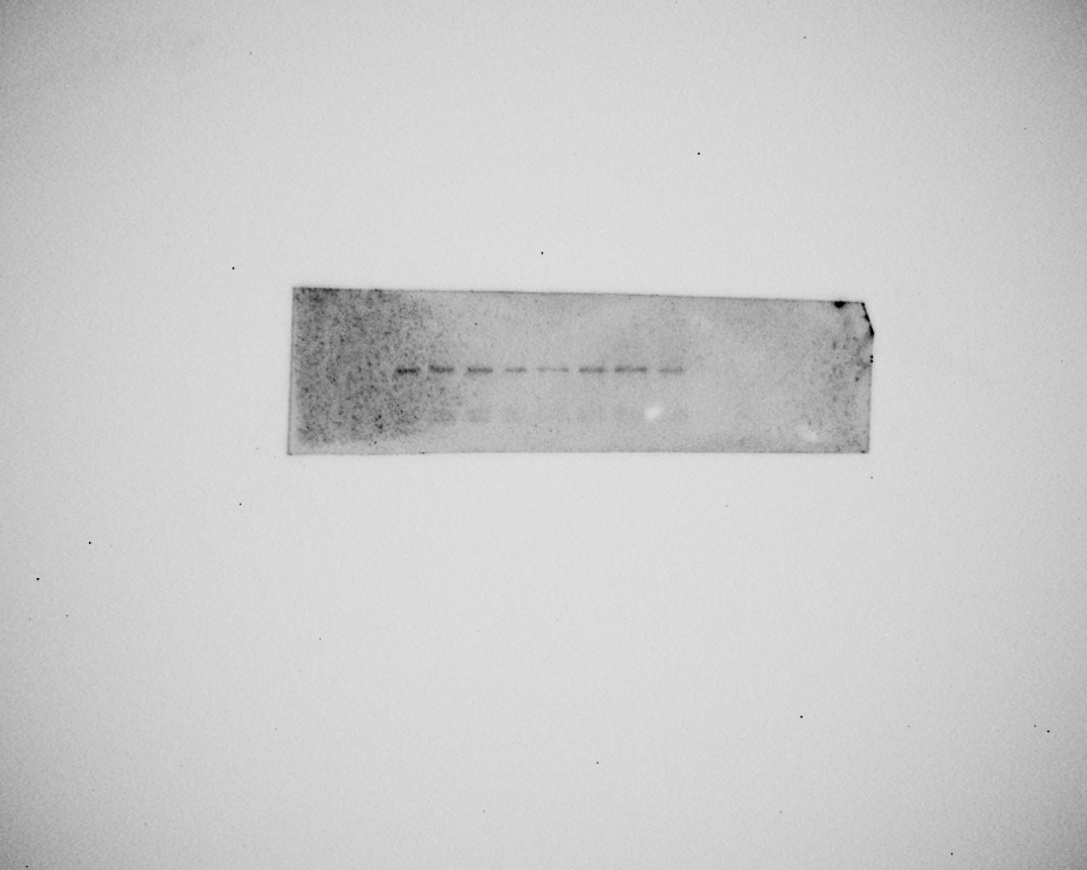


IP:H2A


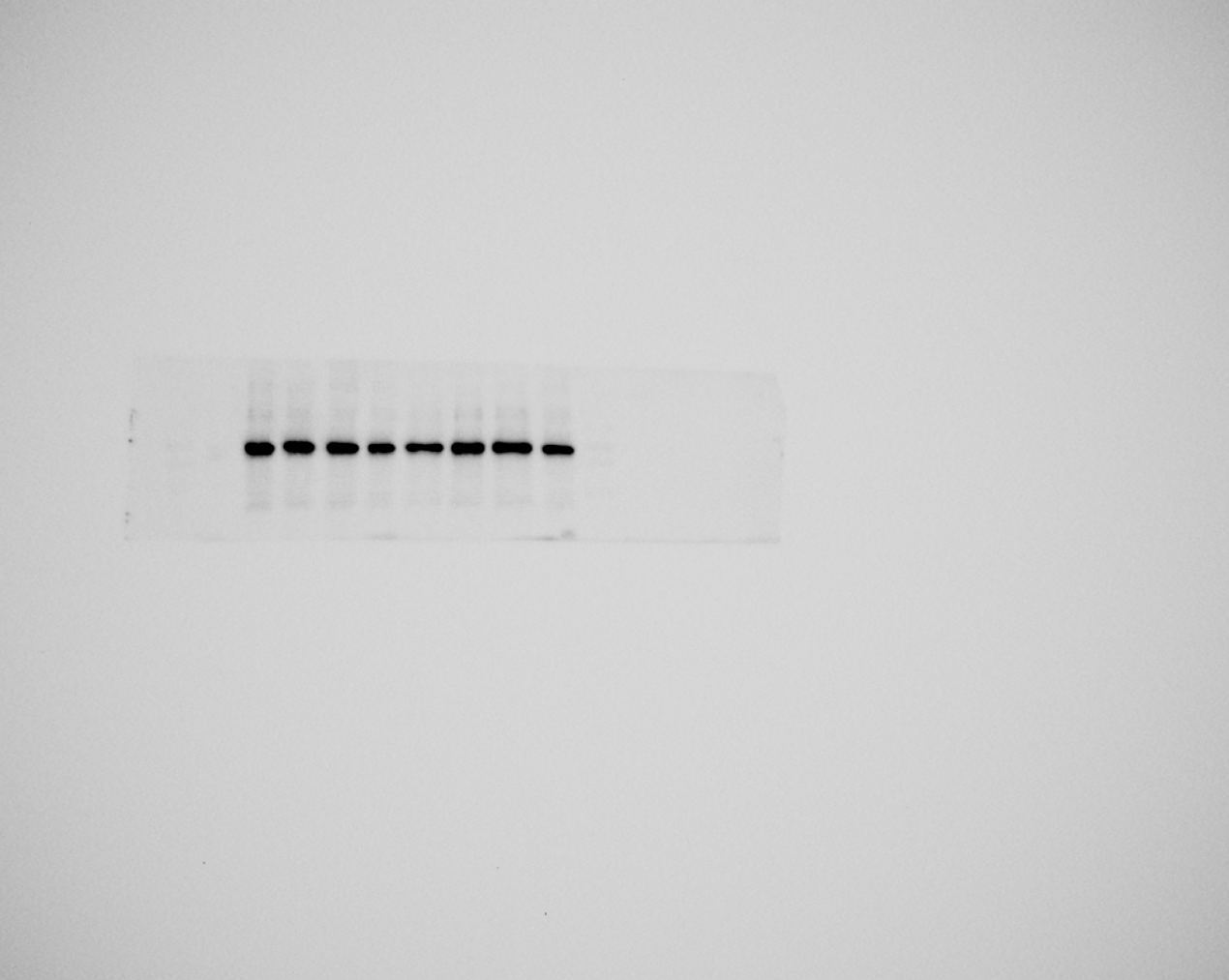


IP:H2AK119ub
